# Key regulatory roles of PRDM1 in human NK-cell differentiation and activation

**DOI:** 10.1038/s41375-025-02815-z

**Published:** 2025-12-09

**Authors:** Xuxiang Liu, Yunfei Shi, Jibin Zhang, Kunal Shetty, Krystie Chew, Can Küçük, Qiang Gong, Esra Esmeray, Haiqing Li, Ru Chen, Sheng Pan, Katarzyna Dąbrowska, Roger E. Moore, Krystine Garcia-Mansfield, Patrick Pirrotte, Jinhui Wang, Yuping Li, Gehong Dong, Logan Lee, Timothy W. McKeithan, Javeed Iqbal, Wing C. Chan

**Affiliations:** 1 Department of Pathology, City of Hope National Medical Center, Duarte, CA 91010 USA; 2 Department of Pathology and Microbiology, University of Nebraska Medical Center, Omaha, NE USA; 3 Key Laboratory of Carcinogenesis and Translational Research (Ministry of Education/Beijing), Department of Pathology, Peking University Cancer Hospital & Institute, Beijing, China; 4 Department of Medical Biology, Faculty of Medicine, Dokuz Eylül University, İzmir, 35340 Türkiye; 5 Basic and Translational Research Program, İzmir Biomedicine and Genome Center, İzmir, 35340 Türkiye; 6 İzmir International Biomedicine and Genome Institute, Dokuz Eylül University, İzmir, 35340 Türkiye; 7 Integrated Genome Core, Beckman Research Institute, City of Hope National Medical Center, Duarte, CA USA; 8 Department of Medicine-Gastroenterology, Baylor College of Medicine, Houston, TX USA; 9 Institute of Molecular Medicine, University of Texas Health Science Center at Houston, Houston, TX USA; 10 Integrated Mass Spectrometry Shared Resource, City of Hope Comprehensive Cancer Center, Duarte, CA 91010 USA; 11 Early Detection and Prevention Division, Translational Genomics Research Institute, Phoenix, Arizona 85004 USA; 12 Department of Pathology, Beijing Tongren Hospital, Capital Medical University, Beijing, 100730 China; 13 Department of Pathology, Beijing Tiantan Hospital, Capital Medical University, Beijing, 100070 China

**Keywords:** Non-hodgkin lymphoma, Lymphocytes, Oncogenes

## Abstract

*PRDM1*, encoding a transcription factor (TF), regulates plasma cell and CD8^+^ T-cell terminal differentiation and T_h_2 lineage specification, while its role in human NK-cell differentiation and homeostasis is largely unknown. Here, we employed a multi-omics approach to dissect the transcriptional control of PRDM1 on human NK-cells. PRDM1 is important in NK-cell terminal differentiation based on gene expression profiling and its targeting of key regulators in the process. *PRDM1*-deleted NK-cells displayed a less mature phenotype simulating the CD56^bright^ NK-cell population accompanied by upregulation of stem-like gene signatures. PRDM1-bound genes were enriched in T/NK-cell receptor signaling, activation, and NK-cell effector functions. PRDM1 could function as a transcriptional repressor as well as an activator as its activities may be modified by association with different TFs and co-factors. The kinetics of its action also varies among its target genes. As a homeostatic factor, PRDM1 is induced upon IL-2 and feeder cell stimulation, but its ability to restrict NK-cell growth upon feeder stimulation may be counteracted by the AP-1-induced transcriptional network. The loss of *PRDM1* activity is frequent in NK-cell malignancies which may lead to decreased homeostatic control, impaired terminal differentiation, enhanced cellular fitness, and the acquisition of more stem-like features, thereby promoting lymphomagenesis.

## Introduction

Natural killer (NK) cells are innate lymphocytes that mediate early immune responses to viral infection and tumor cells by mediating cytolytic activities and producing cytokines to promote inflammation and adaptive immunity. The developmental process and homeostasis of NK-cells are highly regulated, and their dysregulation could cause susceptibility to infection, cancer, and autoimmunity [1–3]. Transcription factors (TFs) are important regulators of NK-cell development, maturation, and homeostasis, among which are ETS-1 [4], PU.1 [5], Ikaros [6], TOX [7], E4BP4 [8] and ID2 [9] in early lineage specification, GATA-3 [10, 11], IRF-2 [12], MEF [13], MITF [14], T-bet, Eomes [15] and PRDM1 [16, 17] in the maturation process and cellular function. Cytokines including the interleukin (IL) family members IL-2/12/15/18/21 play important roles in NK-cell survival, expansion, and effector functions [18–20].

PRDM1 has been characterized as an important transcriptional repressor in lymphoid cells. Terminal differentiation of plasma cells requires PRDM1 [21], which represses *MYC* [22] and genes regulating the B-cell program, *PAX5* [23], *BCL6* [24], *CIITA* [24, 25], *ID3* [24], and *SPIB* [24]. In CD4^+^ T-cells, PRDM1 expression biases T_h_2 over T_h_1 cell differentiation through repression of *IFNG*, *FOS* and *TBX21* [26, 27]. PRDM1 has also been demonstrated to attenuate T-cell proliferation and survival, partly through the downregulation of *IL2* [27]. In CD8^+^ T-cells, PRDM1 controls their terminal differentiation and expression of cytotoxic molecules [28, 29]. A study in tumor infiltrating lymphocytes revealed the transcriptional regulation of co-inhibitory receptors by PRDM1 [30]. PRDM1 has also been shown to be expressed throughout NK-cell development, and different isoforms of PRDM1 can be induced by the cytokines IL-2 and IL-18 [17]. Induction of PRDM1 expression promotes mouse NK-cell maturation, while its knockout impairs maturation but increased proliferative potential [16]. In addition, PRDM1 regulates the expression of cytokines, TNF-α and IFN-γ, in NK-cells [17]. Deletion and loss-of-function mutations of *PRDM1* have been reported in diffuse large B-cell lymphoma [31] and anaplastic large T-cell lymphoma [32]. PRDM1 has also been identified as a tumor suppressor that is frequently inactivated/lost in NK-cell malignancies [33–35].

PRDM1 binds DNA through its zinc finger domain [36] and recruits corepressors to induce epigenetic remodeling and transcriptional silencing. The proline-rich and zinc-finger domains associate with the Groucho corepressor complex [37], histone deacetylase (HDAC) 1 and 2 [38], and a histone methyl transferase (HMT) G9a [39]. In mouse plasma cells, other corepressor complexes were identified to interact with PRDM1, including the Polycomb repressive complex 2 (PRC2), the SWI/SNF (BAF) complex, the nucleosome remodeling deacetylase (NuRD) complex, the nuclear receptor co-repressor (NCoR) complex, and the SIN3 (Swi-independent 3) histone modifying complex [40].

To understand the role of PRDM1 in human NK-cell differentiation and function, we employed a systemic investigation and discovered an extensive network of genes that were bound and regulated by PRDM1 directly or in partnership with other TFs, which were dynamic and changed with different culture conditions.

## Materials and methods

### NK-cell enrichment, culture, and genetic modification

NK-cells were isolated from healthy donors and cultured in X-VIVO15 supplemented by IL-2 or with an engineered K562 feeder cell line. NK-cells are genetically modified using the CRISPR/Cas9 technology (Fig. S1A,B). The details of NK-cell isolation, culture, and genetic modifications are described in Supplemental Materials and Methods (S-M&M).

### ChIP-seq, ATAC-seq, and RNA-seq

Chromatin-Immunoprecipitation sequencing (ChIP-seq) were prepared using the enzymatic ChIP Kit (Cell Signaling, USA, #9005). Assay for transposase-accessible chromatin sequencing (ATAC-seq) samples were prepared using a previously published OMNI ATAC-Seq protocol [41, 42]. RNA sequencing libraries were prepared with KAPA Stranded mRNA Sequence Kit (Kapa Biosystems, USA, #KK8421). For each assay, 3–5 replicates were included for each group based on ENCODE guideline and community standard. Details of the experimental procedures and analyses are described in S-M&M.

### Proteomics study of PRDM1 isoforms and interacting partners

We performed mass spectrometry (MS) to investigate the peptide sequences of PRDM1 isoforms. Rapid immunoprecipitation mass spectrometry of endogenous proteins (RIME) and proximity-based biotinylation with an APEX2 tag were used to identify PRDM1-interacting proteins. Details are described in S-M&M.

### Statistical analyses

All data with error bars are shown as mean ± standard deviation. The Student’s t-test was used for comparison between two groups. Kolmogorov–Smirnov test was used to compare distribution of distance between motifs. Chi-squared test was used for pairwise comparison of proportions. The One-way ANOVA with post hoc Tukey was applied to comparison among multiple groups. Values of *p* < 0.05 or padj < 0.05 were considered significant. The Kolmogorov–Smirnov-like test with pre-ranked gene lists was used for GSEA analysis. A modified Fisher’s test and Benjamini-Hochberg FDR were used for Database for Annotation, Visualization and Integrated Discovery (DAVID) analysis and TOBIAS footprint analysis. Wald test followed by Benjamini-Hochberg correction was used for RNA-seq differential expression analysis. Multiple hypergeometric test was used for motif enrichment analysis in MEME.

## Results

### PRDM1 regulates primary human NK-cell differentiation and activation

**Fig. 1:**
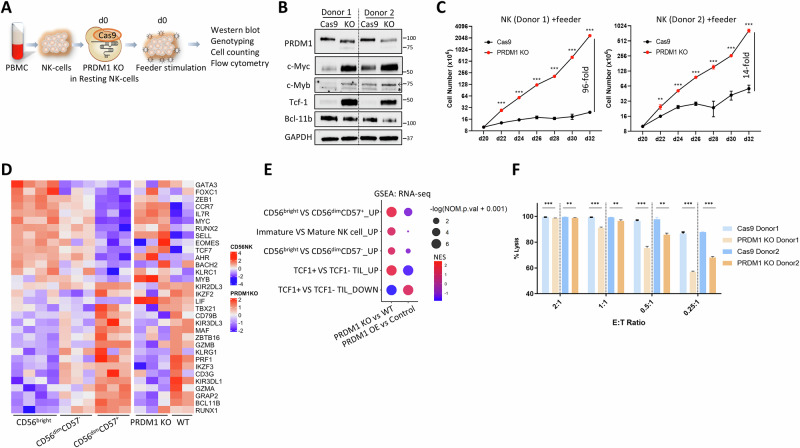
Human NK-cells with *PRDM1* functional KO showed less differentiated and less activated phenotype. **A** Schematic overview of *PRDM1* fKO in human resting NK-cells. **B** Western blotting analysis of PRDM1 and its target genes in *PRDM1*-fKO NK-cells and control cells (Cas9) from two donors. GAPDH was used as a loading control. The arrow indicates the expected band for c-Myb, and the asterisk indicates the non-specific band. **C** Cell counting of *PRDM1*-fKO NK-cells and control cells in late phase of feeder cell stimulation. Feeder cells were added at day 0. **D** Heatmaps showing selected DEGs between *PRDM1*-fKO vs. WT primary human NK-cells that were associated with human NK-cell differentiation from CD56^bright^, CD56^dim^CD57^-^, to CD56^dim^CD57^+^ cells. **E** Dot plot showing significantly enriched pathways from GSEA comparing *PRDM1*-fKO vs. WT NK-cells and *PRDM1* OE (no ASV 12 h) vs. control (+ASV) KHYG1 cells. **F** Cell killing assay measuring NK-cell cytotoxicity when NK-cells were co-cultured with luciferase-expressing K562 target cells overnight at indicated effector:target ratios. Firefly luciferase activity was measured, and percentage of lysis was calculated. For panels **C** and **F** *n* = 3, two independent experiments, two-tailed Student’s t test; *, *p* < 0.05; **, *p* < 0.01; ***, *p* < 0.001.

We previously knocked out (KO) *PRDM1* in primary human NK-cells expanded using a modified K562 cell line expressing membrane-bound IL-21, 4-1BBL, and CD86 (feeder cells) [43]. These NK-cells exhibited faster cell growth with enhanced proliferation, cloning efficiency and reduced apoptosis [44]. The growth advantage acquired from *PRDM1* depletion was recapitulated when the modification was introduced in freshly isolated NK-cells generating fragmental deletion of PRDM1 exon 4 and a shift in protein molecular weight (Fig. 1A–C, Fig. S1C), resulting in disruption of the PR domain that is crucial for the recruitment of transcriptional cofactors to exert its gene regulation function with high KO scores in both donors (termed functional KO, *PRDM1*-fKO).

To further investigate the regulatory role of PRDM1 in human NK-cells, we performed comparative gene expression profiling (GEP) analysis, which indicated that *PRDM1*-fKO NK-cells, compared to wild-type (WT) cells, were more similar to the less differentiated CD56^bright^ NK-cells than the CD56^dim^ counterparts from the peripheral blood [45, 46], with upregulation of *TCF7* (encoding Tcf-1), *SELL*, *IL7R*, *CCR7*, *MYC*, *MYB*, *RUNX2*, and downregulation of *BCL11B*, *IKZF3*, *RUNX1*, *MAF*, and *TBX21* (Fig. 1D). Gene set enrichment analysis (GSEA) confirmed that *PRDM1* fKO resulted in significant enrichment (P < 0.0001) of upregulated genes in CD56^bright^ NK-cells (Fig. 1E, Fig. S1D). Similarly, the GEP of *PRDM1*-fKO NK-cells resembled that of the immature NK-cells rather than the mature cells [47] (Fig. 1E), suggesting a regulatory role of PRDM1 in human NK-cell differentiation. Consistent with the less differentiated phenotype, *PRDM1*-fKO NK-cells showed reduced CD16 expression (Fig. S1E). Notably, *BCL11B*, a master TF that promotes canonical and adaptive NK-cell differentiation [46], was repressed after *PRDM1* fKO (Fig. 1B, D, Fig. S1F). The dysregulation of key genes upon *PRDM1* fKO was confirmed by qRT-PCR (Fig. S1F) and Western blotting (Fig. 1B).

We also employed a regulated system to induce short-term ectopic *PRDM1* expression in an NK-cell lymphoma line, KHYG1, which was sensitive to *PRDM1* expression (Fig. S2A–F). A SMASh [48] tag, containing a degron that is cleavable by a protease, was fused to PRDM1α, and transduced in KHYG1 cells. After withdrawal of the protease inhibitor, Asunaprevir (ASV), PRDM1 proteins accumulated within 6 h (Fig. S2A). qRT-PCR confirmed the expected regulation of several known PRDM1 target genes including *MYC*, *BCL6*, and *IFNG* (Fig. S2B). RNA-seq showed that the differentially expressed genes (DEGs, fold change > 1.5, padj < 0.05) between *PRDM1*-overexpressing (OE) KHYG1 and control cells were significantly enriched (FDR < 0.01) in p53, cell cycle, and T-cell receptor (TCR) signaling pathways (Fig. S2D,E). The DEGs between CD56^bright^ and CD56^dim^ NK-cells were also found to be dysregulated after *PRDM1* short-term OE in KHYG1 cells (Fig. S2F), leading to enrichment of genes associated with NK-cell differentiation (Fig. 1E). We repeated the experiment on primary NK-cells previously expanded in feeder cells and *PRDM1* short-term OE in these NK-cells also led to similar gene expression regulation, displaying varying kinetics for different downstream target genes such as downregulation of *MYC* and upregulation of *IFNG* (Fig. S2G,H).

Concordant with the less mature phenotype, genes associated with NK-cell effector functions including *IFNG*, *TNF*, *FASLG*, *GNLY*, *GZMB*, and *PRF1* were repressed when *PRDM1* was deleted, and all these genes except *GNLY* were upregulated in *PRDM1*-OE KHYG1 cells (Fig. S3A).

Furthermore, GSEA revealed enrichment (P < 0.001) of STEM-like T-cell signature genes [49] in *PRDM1*-fKO cells compared to WT, while *PRDM1* OE in KHYG1 cells led to depletion of STEM-like signatures (Fig. 1E, Fig. S3B). Importantly, master regulators of stem-like features in T-cells, *TCF7* and *MYB* [50], were highly elevated at mRNA and protein levels in *PRDM1*-fKO NK-cells (Fig. 1B, D, Fig. S3B). Consistently, reduction of activation marker CD69 and elevation of stem-like marker CD62L were observed (Fig. S3C). Flow cytometry also confirmed the downregulation of exhaustion marker Tim-3, inflammatory cytokines IFN-γ and TNFα, and effector molecule granzyme B upon *PRDM1* depletion (Fig. S3D,E). The downregulation (P < 0.01) of cytotoxic molecules when *PRDM1* was deleted was associated with diminished NK-cell cytotoxicity (Fig. 1F).

Together, these findings indicate that PRDM1 could be a crucial TF that controls not only homeostatic expansion but also terminal differentiation and effector functions of human NK-cells.

### Different PRDM1 isoforms induced in human NK-cells cultured in IL-2 with or without feeder cells

While IL-2 alone could only maintain human NK-cell survival in vitro with limited proliferation for about a week, NK-cells were able to rapidly proliferate with low apoptosis for more than a month with feeder cells (Fig. S4A). Both IL-2 and feeder cell stimulation induced PRDM1 expression, with the latter exhibiting a more potent effect (Fig. S4B). NK-cells stimulated with feeder cells for 13 days (NK-F-D13) mainly expressed the largest PRDM1 isoform, PRDM1α (Fig. S4B,C), which is considered as the most potent isoform [51]. MS validated the PRDM1α isoform (Fig. S4D). In NK-cells cultured with IL-2 alone for six days (NK-IL2-D6), all isoforms especially the smaller isoforms progressively increased (Fig. S4E), consistent with a previous study [17]. When we continued to culture NK-F-D13 cells for 15 more days without additional feeder cells (NK-F-late), NK-cell proliferation slowed down (Fig. S4A) with increased expression of the smaller PRDM1 isoforms but not PRDM1α (Fig. S4F).

The lowest PRDM1 band likely represented PRDM1β, which is annotated as the shortest isoform 201 by Ensembl. The intermediate PRDM1 isoform had been proposed to be PRDM1α lacking exon 6 (∆exon6) [17]. However, our PCR and RNA-seq analysis could hardly detect PRDM1∆exon6 products from NK-IL2-D6 and NK-F-D13 (Figure S4G).

We overexpressed PRDM1β coding sequence in NKYS cells and detected a strong PRDM1 intermediate band, while the lower band only slightly increased (Fig. S4H). MS study of the intermediate PRDM1 band from these cells identified peptides corresponding to the PRDM1β isoform (Fig. S4I). It is likely that the intermediate band represented a post-translationally modified form of PRDM1β, which shifted the PRDM1β band to a higher molecular weight position.

### PRDM1 binding profiles in human NK-cells

**Fig. 2:**
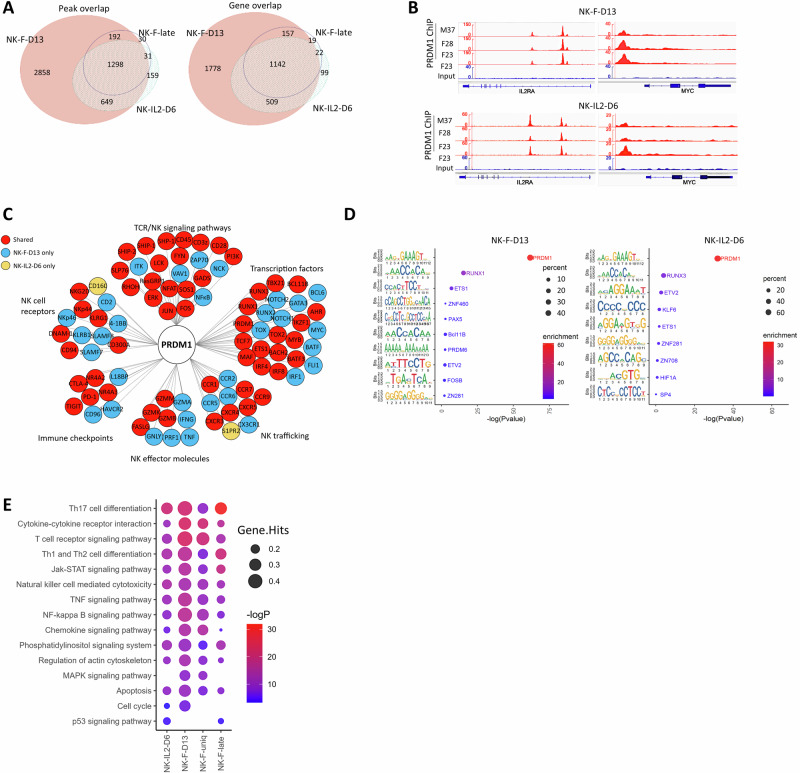
ChIP-seq identified PRDM1 binding spectrum in human NK-cells. **A** Venn diagrams showing the number of overlapping peaks and genes among different groups of samples. **B** Integrative Genomics Viewer (IGV) tracks of PRDM1 ChIP-seq RPKM signals at the *IL2RA* and *MYC* loci. The ChIP-Seq tracks are normalized with RPKM in bamCoverage. **C** Representative PRDM1-bound genes in different categories. **D** Motif enrichment analysis by STREME. *P* value indicates the possibility of identifying the indicated motifs within the peaks randomly. **E** KEGG pathway analysis of PRDM1 bound genes.

**Fig. 3:**
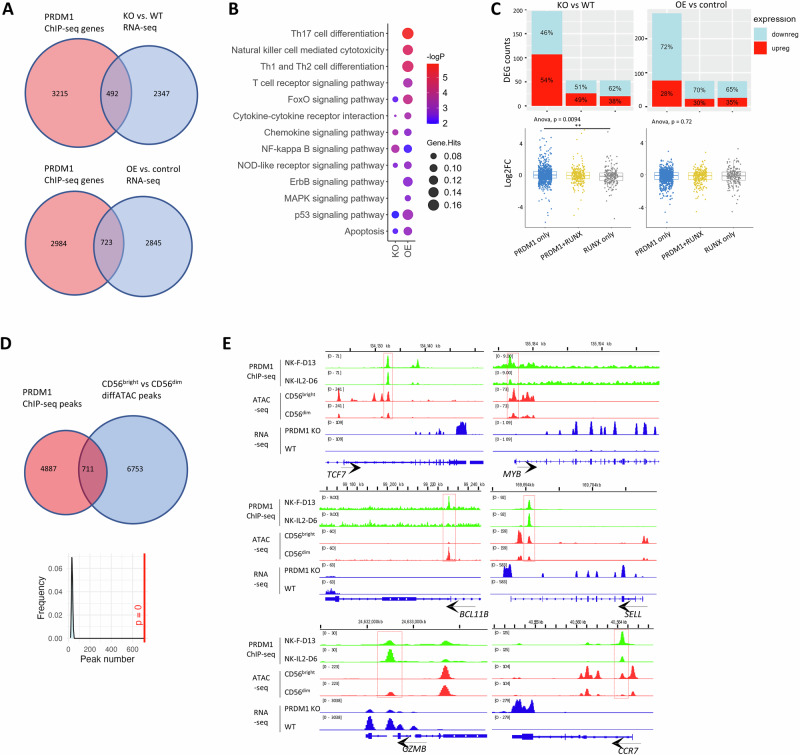
PRDM1 directly bound and regulated key regulatory genes in human NK-cells. **A** Venn diagrams showing the number of overlapping genes between PRDM1-bound genes with DEGs comparing *PRDM1*-fKO and WT NK-cells or comparing *PRDM1* OE and control KHYG1 cells. **B** Pathway analysis of overlapping genes between PRDM1-bound genes and DEGs in RNA-seq. **C** Number and percentage (top) and overall expression levels (bottom) of upregulated and downregulated PRDM1 target genes grouped by PRDM1 and RUNX motifs in *PRDM1*-fKO vs. WT NK-cells or *PRDM1* OE vs. WT KHYG1 cells. ANOVA and post-hoc Tukey test. ** means *p* < 0.01. **D** Overlapping peaks between PRDM1 ChIP-seq and differential ATAC-seq for CD56^bright^ vs. CD56^dim^ NK-cells. The density plot shows overlapping peak numbers distribution in 1000 random shuffle permutation with Bedtools and the red line denotes the observed overlap number and its *p*-value. **E** IGV tracks of selected genes showing PRDM1 binding, differential ATAC-seq between CD56^bright^ and CD56^dim^ NK-cells, and differential expression between *PRDM1*-fKO and WT NK-cells.

We examined the genome-wide PRDM1 binding sites in human NK-cells by ChIP-seq using MACS2 among triplicate samples for each of NK-F-D13, NK-IL2-D6, and NK-F-late groups and identified 4,997, 2,137, and 1,551 peaks respectively present in at least two samples within each group, which are referred to as consensus peaks (Fig. S5A,B). D13 was chosen to ensure all feeder cells were depleted, whereas D6 was chosen for cells cultured without feeder as IL-2 alone was not sufficient to maintain the viability of NK-cells beyond six days. PRDM1 mainly bound intergenic, intronic, and promoter regions (Fig. S5C). Notably, ~80% of PRDM1-bound peaks were found within the promoter (±1KB of TSS) or at the enhancer and super enhancer regions identified by Homer through H3K27ac sequencing analysis of NK cells in the same culture conditions (Fig. S5D). Principal component analysis (PCA) showed that samples from different culture conditions were well separated (Fig. S5E). The consensus peaks were used to identify 1,772 and 3,586 PRDM1-bound genes in NK-IL2-D6 and NK-F-D13, respectively, with 93.2% (1,651/1,772) of genes in NK-IL2-D6 shared with those in NK-F-D13 (Fig. 2A). Importantly, some of the previously identified PRDM1 target genes, mostly in mouse B- and T-cells, were also detected by our studies, such as *IL2RA* [52], *MYC* [22], and *TCF7* [53] (Fig. 2B, C, Fig. S5F). Binding motif analysis by MEME-ChIP identified PRDM1 as the top consensus motif with high confidence (P < 10^-25^) (Fig. 3D). The RUNX family binding motifs and other TF motifs were also detected with differential enrichment (*P* < 0.0001) between NK-IL2-D6 and NK-F-D13 (Fig. 3D). In NK-IL2-D6, 59.3% and 9.7% of PRDM1 ChIP-seq peaks contained PRDM1 only and RUNX only motif respectively, while 17.1% of the peaks harbored both motifs. In contrast, NK-F-D13 showed increased percentage of the RUNX and other motifs, and this is especially significant (*P* < 0.01) for peaks unique to NK-F-D13 (NK-F-uniq) in Chi-squared test (Fig. S5G). The peak/gene profile and the motif enrichment pattern of NK-F-late resembled those of NK-IL2-D6 (Fig. 2A, Fig. S5G). The distance between PRDM1 and RUNX motifs was mainly around 25 bp when both were detected within a PRDM1-bound peak (Fig. S5H).

### PRDM1 binds and regulates genes associated with NK-cell differentiation, activation, and function

PRDM1-bound genes were enriched in pathways associated with lymphocyte activation and functions, such as Th17 cell differentiation, cytokine-cytokine receptor interaction, TCR signaling pathway, and NK-cell mediated cytotoxicity, with greater enrichment in NK-F-D13, while NK-IL2-D6 and NK-F-late showed enrichment in p53 signaling pathway in DAVID pathway analysis (Fig. 2E).

The antigen receptor signaling pathways in T- and B- cells share many components with NK-cells. PRDM1-bound genes included a large number of genes in the TCR signaling pathway and those involved in NK-cell activation and NK-cell mediated cytotoxicity (Fig. 2C, E, Fig. S6A–D). PRDM1 targeted various NK-cell receptors and also bound genes of the signaling lymphocytic activation molecule (SLAM) family (Fig. 2C), which are crucial for regulating NK-cell activation and subsequent effector programs when encountering pathogens or tumor cells and for restraining the effector functions upon ligation to MHC class I molecules [54]. PRDM1 also bound genes encoding immune checkpoint molecules, interleukin receptors, cytokines, NK-cell cytotoxicity effectors, chemokine receptors and ligands responsible for NK-cell trafficking, TFs associated with NK-cell development and differentiation, and apoptosis-regulating genes (Fig. 2C).

To identify PRDM1-bound genes that are functionally relevant in human NK-cells, we integrated the PRDM1 ChIP-seq data with RNA-seq data. 492 and 723 PRDM1-bound genes were DEGs in the *PRDM1*-fKO vs. WT NK-cell and *PRDM1*-OE vs. control KHYG1 cell comparison, respectively (Fig. 3A). In DAVID KEGG pathway analysis, these PRDM1-bound DEGs were enriched in Th17 cell differentiation, NK-cell mediated cytotoxicity, TCR, and p53 signaling pathways comparing *PRDM1*-OE vs. control KHYG1 cells, and in chemokine and NF-kappa B signaling pathways comparing *PRDM1*-fKO vs. WT NK-cells (Fig. 3B).

Motif analysis demonstrated a trend that target genes with only PRDM1 motif were more likely to be upregulated and showed higher (*P* < 0.01) expression levels upon *PRDM1* fKO compared with those with RUNX only motif (Fig. 3C, left panel). In contrast, short-term *PRDM1* OE led to repression of most of the PRDM1 target genes, with a slightly higher percentage of upregulated genes and increased expression when only RUNX motifs were present (Fig. 3C, right panel). These findings suggested that PRDM1 mainly acts as a transcriptional repressor when binding directly to targets and that the association with RUNX or other TFs may lead to more divergent regulation of target gene expression.

By overlapping our PRDM1 ChIP-seq peaks with differentially accessible ATAC-seq regions between CD56^bright^ and CD56^dim^ NK-cells [45], we identified 711 PRDM1-bound sites that displayed changes in chromatin accessibility during CD56^bright^ to CD56^dim^ NK-cell transition (Fig. 3D). Many of these differentially accessible PRDM1-bound peaks were associated with differential gene expression between *PRDM1*-fKO and WT NK-cells, including the key regulators of stem-like features (*TCF7*, *MYB*), master TF that controls NK cell terminal differentiation (*BCL11B*), NK effector molecule (*GZMB*), and stem memory markers (*SELL*, *CCR7*) (Fig. 3E). These results strongly suggest that PRDM1 binding may directly regulate chromatin accessibility and transcription of important target genes involved in human NK-cell differentiation, activation, and stem-like phenotype acquisition.

### Chromatin accessibility in stimulated human NK-cells and association with PRDM1 binding

**Fig. 4:**
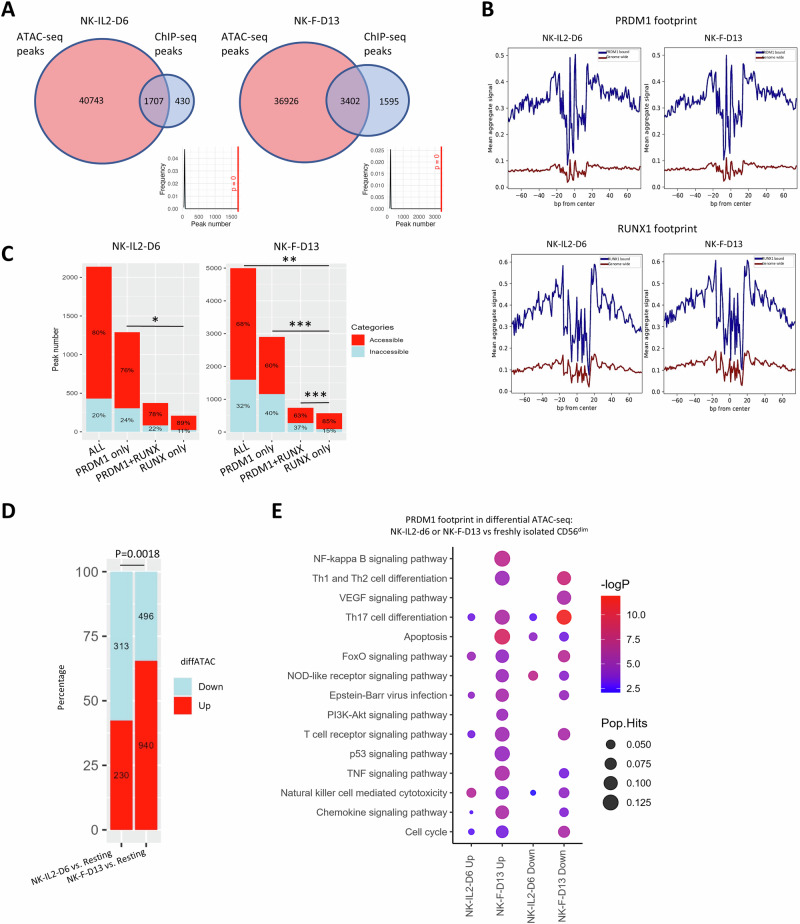
ATAC-seq analysis identified chromatin accessibility status that may be associated with PRDM1 binding in human NK-cells. **A** Overlapping peaks between ATAC-seq and PRDM1 ChIP-seq. The density plots show distribution of overlapping peak numbers in 1000 random shuffle permutations with Bedtools and the red lines denote the observed overlap numbers and their *p*-values. **B** ATAC-seq signals showing PRDM1 (top) and RUNX (bottom) footprints in NK-IL2-D6 and NK-F-D13. **C** Bar graph showing the number and percentage of PRDM1-bound sites with PRDM1 and/or RUNX motifs that were accessible or inaccessible. *P*-value indicates the significance of Chi-squared test of percentage between two groups. *, *p* < 0.05; **, *p* < 0.01; ***, *p* < 0.001. Bar graph showing number and percentage **D** and KEGG pathway analysis **E** of PRDM1 footprint-containing genes that showed differential ATAC signal between NK-IL2-d6 or NK-F-D13 vs. freshly isolated CD56^dim^ NK-cells. *P* value indicates significance in Chi-squared test of the proportion.

We performed ATAC-seq on NK-IL2-D6 and NK-F-D13 and identified a total of 42,450 and 40,328 ATAC peaks, respectively (Fig. 4A). The replicate samples clustered together (Fig. S7A). Significantly enriched (*p* < 10^-150^) STAT5B footprints were identified in the accessible regions in both NK-IL2-D6 and NK-F-D13 compared to resting NK-cells [45] (Fig. S7B), consistent with the activation of JAK-STAT5 pathway with IL-2 stimulation. Importantly, both PRDM1 and RUNX motif footprints were also significantly enriched (*p* < 10^-120^) after IL-2 or feeder cell stimulation, indicating that these two TFs are important in the TF network in NK-cells upon activation (Fig. 4B, Fig. S7B).

20.1% (430/2137) and 31.9% (1595/4997) of PRDM1-bound sites from ChIP-seq data were inaccessible (closed chromatin regions, CCRs) in NK-IL2-D6 and NK-F-D13, respectively (Fig. 4A). NK-F-D13 had a higher percentage of PRDM1-bound CCRs in each motif category (Fig. 4C). Notably, PRDM1-bound sites with only PRDM1 motif showed a higher percentage of CCRs, whereas those with only RUNX motif have significantly higher percentage (*P* < 0.05) of accessible regions in both NK-cell culture conditions (Fig. 4C), suggesting that direct PRDM1 binding to consensus motif resulted more often in transcriptional repression compared with those with RUNX motifs. Therefore, PRDM1 binding does not always associate with closed chromatin, and the chromatin accessibility status is established collectively by PRDM1, associated TFs, and cofactors.

To interrogate the potential functional effects of PRDM1-associated chromatin accessibility changes on NK-cells upon stimulation, we compiled the genes with PRDM1 footprint that were differentially accessible between NK-IL2-D6 or NK-F-D13 and unstimulated NK-cells for pathway analysis (Fig. 4D, E). Compared with resting NK-cells, NK-IL2-D6 accessible genes with PRDM1 footprint were enriched in NK-cell mediated cytotoxicity pathway. In NK-F-D13, PRDM1 footprint-containing genes with higher ATAC-seq signal were enriched in the apoptosis pathway, while the less accessible genes were enriched in the Th17 differentiation pathway, suggesting that PRDM1 may regulate chromatin accessibility of different sets of genes after IL-2 or feeder cell stimulation.

### AP-1 factors may counteract the repressive function of PRDM1 in feeder-stimulated NK-cells

Next, we utilized RNA-seq and ATAC-seq to dissect the differences between NK-cells stimulated with feeder cells and cultured with IL-2 alone. RNA-seq revealed that proliferation-related pathways were more enriched in NK-F-D13 than in NK-IL2-D6 (Fig. S8A–C). *CD86* was significantly induced upon feeder stimulation (Fig. S8D), concordant to the induced expression of *CD86* in NK-cells treated with IL-2 and IL-21 [55].

**Fig. 5:**
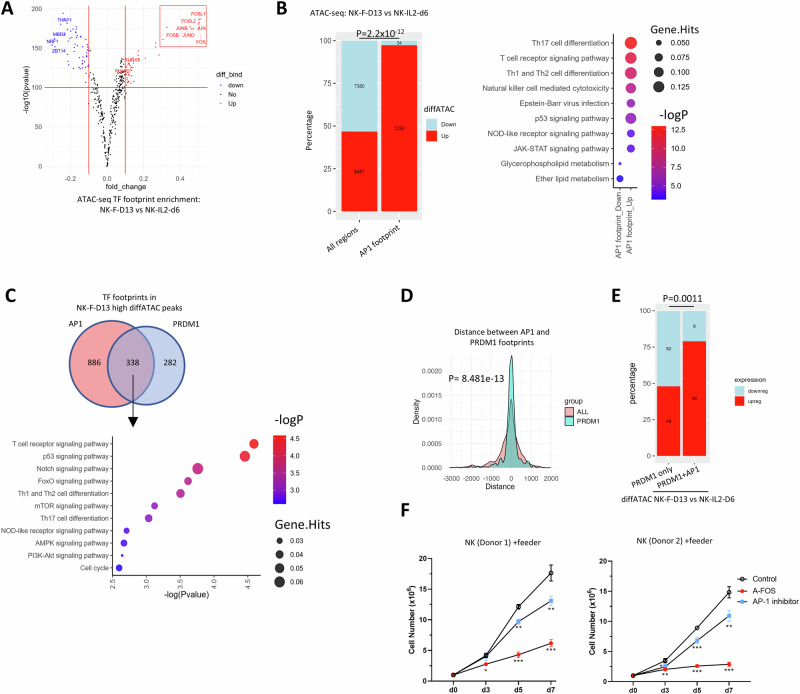
Homeostatic control of NK-cells by PRDM1 may be counteracted by AP-1 factors when stimulated with feeder cells. **A** Volcano plots showing significantly enriched TF footprints in ATAC-seq comparing NK-F-D13 with NK-IL2-D6. **B** Number and percentage (left) and pathway analysis (right) of all differential ATAC-seq peaks and AP-1 footprint-containing differential ATAC-seq peaks comparing NK-F-D13 and NK-IL2-D6. *P* value on the bar graph indicates significance in Chi-squared test of the proportion. **C** Number (top) and pathway analysis (bottom) of genes with both AP-1 and PRDM1 footprint that showed higher ATAC-seq signal in NK-F-D13 compared to NK-IL2-D6. **D** Distance between AP-1 and PRDM1 footprints or any footprints within differential ATAC-seq peaks between NK-F-D13 and NK-IL2-D6. *P*-value indicates the significance of Kolmogorov–Smirnov test of the two distributions. **E** Number and percentage of DEGs between NK-F-D13 and NK-IL2-D6 among genes with differential ATAC-seq signals between NK-F-D13 and NK-IL2-D6 containing PRDM1 footprint only or both PRDM1 and AP-1 footprints. *P* value indicates significance in Chi-squared test of the proportion. **F** Cell counting of primary human NK-cells transduced with A-FOS or treated with 5 μM AP-1 inhibitor T5224 after feeder cell stimulation in two independent experiments with 3 replicates. Comparison with control group as reference were performed by one-way ANOVA following Tukey ad-hoc test at each time point. *, *p* < 0.05; **, *p* < 0.01; ***, *p* < 0.001.

Strikingly, NK-F-D13 showed a marked increase (*P* < 10^-150^) in AP-1 factor (*JUN*, *JUNB*, *FOS*, *FOSB*, *FOSL1* and *FOSL2*) footprints (Fig. 5A) compared with NK-IL2-D6 in TOBIAS BINDetect analysis, suggesting that AP-1 factors play a crucial role in NK-F-D13 functional characteristics. We also identified FOSL1 as the top enriched motif within sites that were more accessible in NK-F-D13 than in NK-IL2-D6 (Fig. S8E) through MEME motif enrichment test. In addition, 97.3% (1224/1258) of the AP-1 footprint containing sites were more accessible in NK-F-D13, in contrast to overall less than 50%, with enrichment in the Th17 differentiation, TCR signaling pathway, and apoptosis pathway (Fig. 5B). Transcription factor activity scored based on weighted mean of target genes expression in RNA-seq in decoupleR showed proliferation-associated TFs (MYC and E2Fs) and AP-1 factors (JUNB, JUN, AP-1) among the top TFs that were important in the regulatory networks in NK-F-D13 (Fig. S8F). These findings collectively suggest that AP-1 factors strongly promote chromatin accessibility and upregulate genes that are important in NK-cell activation and function upon feeder cell stimulation.

Next, we asked whether AP-1 targets had a significant overlap with PRDM1 targets. Indeed, 54.5% (338 of 620) of PRDM1 footprint-containing sites also harbored AP-1 footprint in NK-F-D13, and these genes were enriched in TCR and p53 signaling pathways (Fig. 5C). The significantly (*P* < 10^-12^) short distance between AP-1 and PRDM1 footprints compared to all footprints tested within these sites (Fig. 5D) in Kolmogorov–Smirnov test indicated that the binding of AP-1 factors may modulate PRDM1-mediated transcriptional repression. Indeed, compared to the sites with PRDM1 footprint only, the presence of adjacent AP-1 footprint was associated with drastically higher (*P* < 0.01) percentage of upregulated genes in Chi-squared test (Fig. 5E). Therefore, although NK-F-D13 expressed the potent form of PRDM1, they were able to proliferate rapidly possibly due to the strong activating effects of the AP-1 factors.

To further examine the effects of AP-1 factors in promoting NK-cell growth with feeder cell stimulation, we inhibited the activity of AP-1 by transducing primary NK-cells with a dominant-negative form of AP-1, A-FOS [56], or treating with an AP-1 inhibitor, T5224, both of which induced significant growth suppression (Fig. 5F, S9A–C). Therefore, AP-1 factors are required to induce NK-cell growth in response to feeder cell stimulation.

### Mass spectrometric analysis of PRDM1 binding partners in human NK-cells

**Fig. 6:**
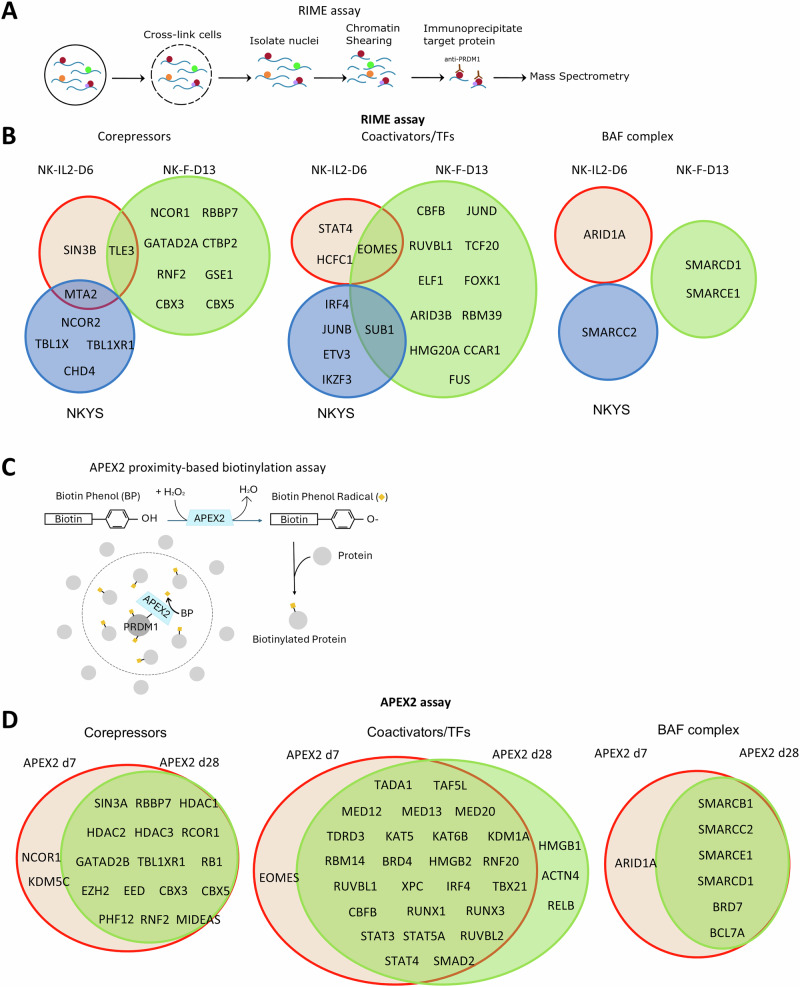
Proteomic analysis of PRDM1-associated proteins. **A** Schematic overview of the RIME assay. **B** RIME assay identified transcription cofactors associated with PRDM1 in primary human NK-cells or in NKYS cells, which are grouped into corepressors, coactivator/transcription activators, and BAF complex components. The dataset from IgG control group was used to remove nonspecific background. **C** Schematic overview of the APEX2 proximity-based biotinylation assay. **D** PRDM1-associated cofactors identified by the APEX2 experiment. Proteins with more than two-fold increase of the normalized peptide count in APEX2 knock-in samples compared to Cas9 control were shown.

To identify chromatin-bound PRDM1-interacting proteins, we employed the RIME assay on NK-IL2-D6, NK-F-D13, and the NKYS cell line (Fig. 6A). We detected previously reported PRDM1-associated corepressor components, including hGroucho3 (encoded by TLE3), SIN3B in the SIN3-corepressor complex, NCoR1/NCoR2/TBL1X in the NcoR complex, RBBP7 in the NuRD complex, and ARID1A/SMARCD1/SMARCE1 in the BAF chromatin remodeling complex (Fig. 6B). Novel binding partners such as CTBP2 in the CoREST complex and TFs that may be responsible for transcriptional activation (e.g., coactivators HCFC1 and SUB1, TFs EOMES and JUNB) were also identified. Importantly, CBF-β, an essential binding partner of the RUNX family members, was detected in NK-F-D13, consistent with the enrichment of RUNX1 motif in PRDM1-bound regions (Fig. 2D).

As RIME assay may not detect weak or transient protein-protein interactions, we also employed the APEX2 proximity-based biotinylation method, which enables efficient biotin-labeling of proteins within ~20 nm radius [57]. NK-cells expressing APEX2-tagged PRDM1 were cultured with feeders for 7 or 28 days for the analysis of proximity-labeled proteins (Fig. 6C, S10A–C). Of the 30 PRDM1-associated cofactors identified by RIME, 12 (40%) were also detected in the APEX2 assay (Fig. S10D). Consistent with the RIME assay, RUNX3, RUNX1, and CBF-β were also biotinylated by PRDM1-APEX2 (Fig. 6D). A lot more cofactors were identified in the APEX2 experiment, including HDAC1/2, histone deacetylases present in multiple corepressor complexes, similar to a previous study in mouse plasmablasts [40]. Western blotting confirmed the identification of corepressors (NcoR1 and SIN3B) and TFs (EOMES and CBF-β), and also identified TLE3 and G9a, which were not detected by MS (Fig. S10E). However, there were not significant differences between the associated cofactors from the d7 and d28 time points after feeder stimulation, which may be partly due to a PRDM1α-dominant effect, as only PRDM1α was detected as a strong band fused with APEX2 (Fig. S10E). Together, the proteomic study identified novel and previously reported PRDM1-associated proteins that might be crucial for PRDM1 to exert its function.

## Discussion

*PRDM1* plays a critical role in B-cell differentiation to antibody secreting cells and in the terminal differentiation of CD8^+^ T-cells. There is evidence that *Prdm1* regulates early NK-cell development and maturation in the mouse [16]. A systemic study in human NK-cells suggests a reciprocal transcriptional program that orchestrates the conversion between human CD56^bright^ and CD56^dim^ cells, involving *TCF1*/*LEF1*, *BACH2*, *PRDM1*, and *MAF* [45]. Another study on human NK-cells identified *BCL11B* as a crucial regulator of conventional and memory NK-cell differentiation [46]. Our study provided evidence that *PRDM1* is a key factor regulating CD56^bright^ to CD56^dim^ NK-cell transition by directly targeting key TFs, such as *TCF7* and *BCL11B*, which is associated with changes in chromatin accessibility at the PRDM1-bound sites and in RNA expression. *PRDM1*-fKO NK cells acquired a more stem-like gene signature that could induce a less differentiated, more stem-like phenotype and may promote lymphoma development. *PRDM1* fKO was also associated with downregulation of effector functions including cytotoxic molecules and cytokines such as *IFNG* and *TNFA*. Smith et al. reported derepression of the two cytokines when *PRDM1* was knocked down [17]. The difference could have resulted from different culture conditions (feeder vs. cytokine stimulation), gene modification methods (knockdown vs. fKO) as partial PRDM1 knockdown may lead to its upregulation due to the negative auto-regulatory loop of PRDM1, and assay time point (steady state after *PRDM1* fKO vs. 48 h after *PRDM1* KD).

ChIP-seq analysis demonstrated that PRDM1 target spectra varied widely depending on the culture conditions. While PRDM1 motif was the dominant motif, other TF motifs, particularly the RUNX motifs, were also detected, and their abundance also varied with the culture conditions, which indicates that the differential association of TFs with PRDM1 could modify PRDM1 function. PRDM1 targets were highly enriched in the TCR signaling and T-cell activation pathway, as well as in pathways associated directly with NK-cell activation, effector functions, and migration, strongly supporting the crucial role of PRDM1 as a regulator of NK-cell function. The “Th17 differentiation pathway” was highly enriched in many of the pathway analyses. However, many of the enriched genes were not specific to Th17 differentiation per se, including genes in NFAT-AP1, JAK-STAT, NF-kb, and TGFβ pathways. The TGFβ pathway has been reported to regulate NK-cell activation, functions, and plasticity [58]. Thus, this pathway is more related to T/NK-cell activation and development than Th17 differentiation.

PRDM1 isoform expression appears to be correlated with the levels of stimulation with strong stimuli like the feeder cells inducing the expression of the more potent PRDM1α isoform to confer stronger inhibitory effects. However, it seems paradoxical that NK-cells were able to escape the growth inhibition by PRDM1 when cultured with feeder cells despite the expression of more potent PRDM1α isoform. There is evidence showing PRDM1 is still functional in NK-cells cultured with feeder cells: NK-F-D13 exhibited (1) large number of PRDM1 binding peaks (Fig. 2A) and (2) higher percentage of closed chromatin than in NK-IL2-D6 (Fig. 4C); (3) *PRDM1* fKO further elevated NK-cell growth with feeder stimulation (Fig. 1C) [44]. NK cells were still able to grow despite the presence of PRDM1 activities possibly because feeder stimulation provides strong activation signals that overcome the inhibition. In this context, we noticed enriched AP-1 factor footprint in NK-F-D13 compared to NK-IL2-D6 cells. AP-1 factors have been shown to be essential for the development, homeostasis, and effector functions of NK-cells [59]. Our study demonstrated the enrichment of AP-1 footprints in TCR signaling pathway, their significant overlap with PRDM1 footprints, and the close proximity between AP-1 and PRDM1 footprints. These collectively suggest a possible role of AP-1 in activating its target genes, nullifying the influence of repressors if present. However, it is not conclusively proven that AP-1 factors directly counteracted the PRDM1-induced repression, and this is a strong hypothesis based on the evidence we obtained. The regulation of NK-cell homeostasis by AP-1 when grown with feeder cells was validated through the inhibitory effect induced by a dominant negative A-FOS construct and to a lesser extent by a small molecule inhibitor (Fig. 5F).

Different RUNX proteins are expressed at different stages of NK-cell lineage specification, differentiation, and maturation [46, 60, 61]. The interaction between PRDM1 and RUNX in mature NK-cells suggests the modulation of PRDM1 activities in the regulation of NK-cell differentiation and function by RUNX family members. Other TF binding motifs were also detected but at much lower frequencies. Some of these TFs were detected by RIME and/or APEX2 experiments (EOMES, IRF4, and STAT family members) which also detected multiple co-repressors, co-activators and members of the BAF complex. These factors were probably present in different complexes that modulated PRDM1 activities resulting in changes in chromatin architecture with or without concomitant changes in transcriptional activities. Indeed, we found that PRDM1 re-expression in KHYG1 cells upregulated some of the PRDM1 bound genes in a time-dependent manner. However, further mechanistic studies are required to prove and elucidate how PRDM1 could promote transcriptional activation. Interestingly, a previous study demonstrated the binding of STAT3 to the PRDM1 locus to increase its expression [62]. We previously identified the functional cooperation between PRDM1 loss of function alterations and STAT3 mutations in the regulation of NK-cell growth and oncogenic transformation [63]. Deletion of PRDM1 could prevent the induction of its expression from hyperactive STAT3 proteins, resulting in disruption of this negative feedback loop and release of the oncogenic effect of STAT3 mutants. Perturbation of the physical interaction between STAT3 and PRDM1 could also underlie the co-occurrence of PRDM1 loss and STAT3 mutations in NK-cell lymphomagenesis as PRDM1 could be recruited to STAT3 target gene loci to terminate their expression by recruiting co-repressor complexes. T-bet and EOMES have been shown to control the functions and identity of NK-cells [64, 65], in line with the regulation of NK-cell differentiation by PRDM1.

We have provided strong evidence on the role of PRDM1 in regulating NK-cell terminal differentiation and as a regulator of NK-cell growth and activation. We have demonstrated that PRDM1 targeted a large number of genes involved in T/NK cells activation/signaling, trafficking and effector functions, and identified a number of targets genes and pathways that are important for PRDM1 to mediate its function. We also confirmed known and identified novel corepressors and coactivators/TFs as PRDM1 binding partners, which may determine whether it represses or activates the target genes. AP1 factors are highly active in NK-cells grown in feeder and may modulate the repressive activity of PRDM1. Further studies are needed to decipher how different stimuli would influence PRDM1 and its isoform expression, PRDM1/TF interactions, and subsequent co-repressor or co-activator recruitment.

## Supplementary information

Supplementary Figures

Supplementary Figure and Table Legend

Supplementary Tables

Supplemental Material and Methods

## Data Availability

The next generation sequencing data are deposited at https://www.ncbi.nlm.nih.gov/sra/PRJNA1121024. The mass spectrometry proteomics data have been deposited to the ProteomeXchange Consortium via the PRIDE partner repository with the dataset identifier PXD068794 and PXD069044. The data generated in this study are available upon request from the corresponding author.

## Code availability

The code for this project is freely accessible on GitHub at https://github.com/jibzhang/PRDM1_multiomics_in_NK.
