# Supplementary material for: Key regulatory roles of PRDM1 in human NK-cell differentiation and activation
Source: Leukemia. 2025 Dec 9;40(1):199–210. doi: 10.1038/s41375-025-02815-z (PMC12789010; doi:10.1038/s41375-025-02815-z)
Supplement: Supplementary file 1 — Supplementary Figures [file 41375_2025_2815_MOESM1_ESM.pdf]

Figure S1

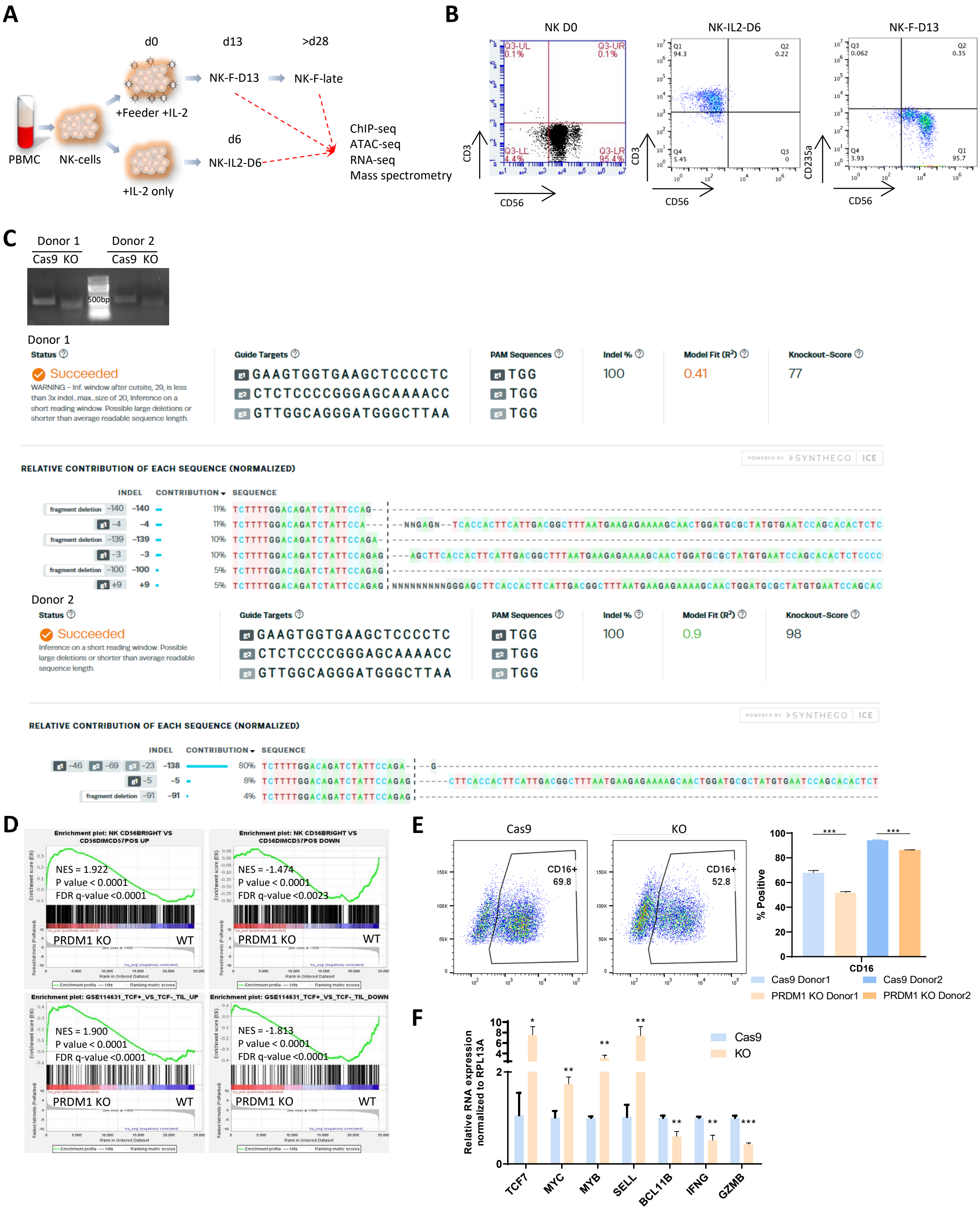
A
d0
d13
>d28
NK-F-D13
NK-F-late
+Feeder +IL-2
d6
NK-IL2-D6
PBMC
NK-cells
+IL-2 only
ChIP-seq
ATAC-seq
RNA-seq
Mass spectrometry
B
NK D0
NK-IL2-D6
NK-F-D13
Q3-UL 0.1%
Q3-UR 0.1%
Q3-LL 4.4%
Q3-LR 95.4%
Q1 94.3
Q2 0.22
Q4 5.45
Q3 0
Q3 0.062
Q2 0.35
Q4 3.93
Q1 95.7
CD3
CD235a
CD56
C
Donor 1
Donor 2
Cas9 KO
Cas9 KO
500bp
Donor 1
Status
Succeeded
WARNING - Inf. window after cutsite, 29, is less than 3x indel_max_size of 20, Inference on a short reading window. Possible large deletions or shorter than average readable sequence length.
Guide Targets
GAAGTGGTGAAGCTCCCCTC
CTCTCCCCGGGAGCAAAACC
GTTGGCAGGGATGGGCTTAA
PAM Sequences
TGG
TGG
TGG
Indel %
100
Model Fit (R²)
0.41
Knockout-Score
77
RELATIVE CONTRIBUTION OF EACH SEQUENCE (NORMALIZED)
INDEL
CONTRIBUTION
SEQUENCE
fragment deletion -140 -140 11%
-4 -4 11%
fragment deletion -139 -139 10%
-3 -3 10%
fragment deletion -100 -100 5%
+9 +9 5%
Donor 2
Status
Succeeded
Inference on a short reading window. Possible large deletions or shorter than average readable sequence length.
Guide Targets
GAAGTGGTGAAGCTCCCCTC
CTCTCCCCGGGAGCAAAACC
GTTGGCAGGGATGGGCTTAA
PAM Sequences
TGG
TGG
TGG
Indel %
100
Model Fit (R²)
0.9
Knockout-Score
98
RELATIVE CONTRIBUTION OF EACH SEQUENCE (NORMALIZED)
INDEL
CONTRIBUTION
SEQUENCE
-46 -69 -23 -138 80%
-5 -5 8%
fragment deletion -91 -91 4%
POWERED BY SYNTHEGO ICE
D
Enrichment plot: NK CD56BRIGHT VS CD56DIMCD57POS UP
NES = 1.922
P value < 0.0001
FDR q-value <0.0001
PRDM1 KO
WT
Enrichment plot: NK CD56BRIGHT VS CD56DIMCD57POS DOWN
NES = -1.474
P value < 0.0001
FDR q-value <0.0023
PRDM1 KO
WT
Enrichment plot: GSE114631_TCF+_VS_TCF-_TIL_UP
NES = 1.900
P value < 0.0001
FDR q-value <0.0001
PRDM1 KO
WT
Enrichment plot: GSE114631_TCF+_VS_TCF-_TIL_DOWN
NES = -1.813
P value < 0.0001
FDR q-value <0.0001
PRDM1 KO
WT
E
Cas9
KO
CD16+ 69.8
CD16+ 52.8
% Positive
CD16
***
***
Cas9 Donor1
Cas9 Donor2
PRDM1 KO Donor1
PRDM1 KO Donor2
F
Relative RNA expression normalized to RPL13A
Cas9
KO
TCF7
MYC
MYB
SELL
BCL11B
IFNG
GZMB

Figure S2

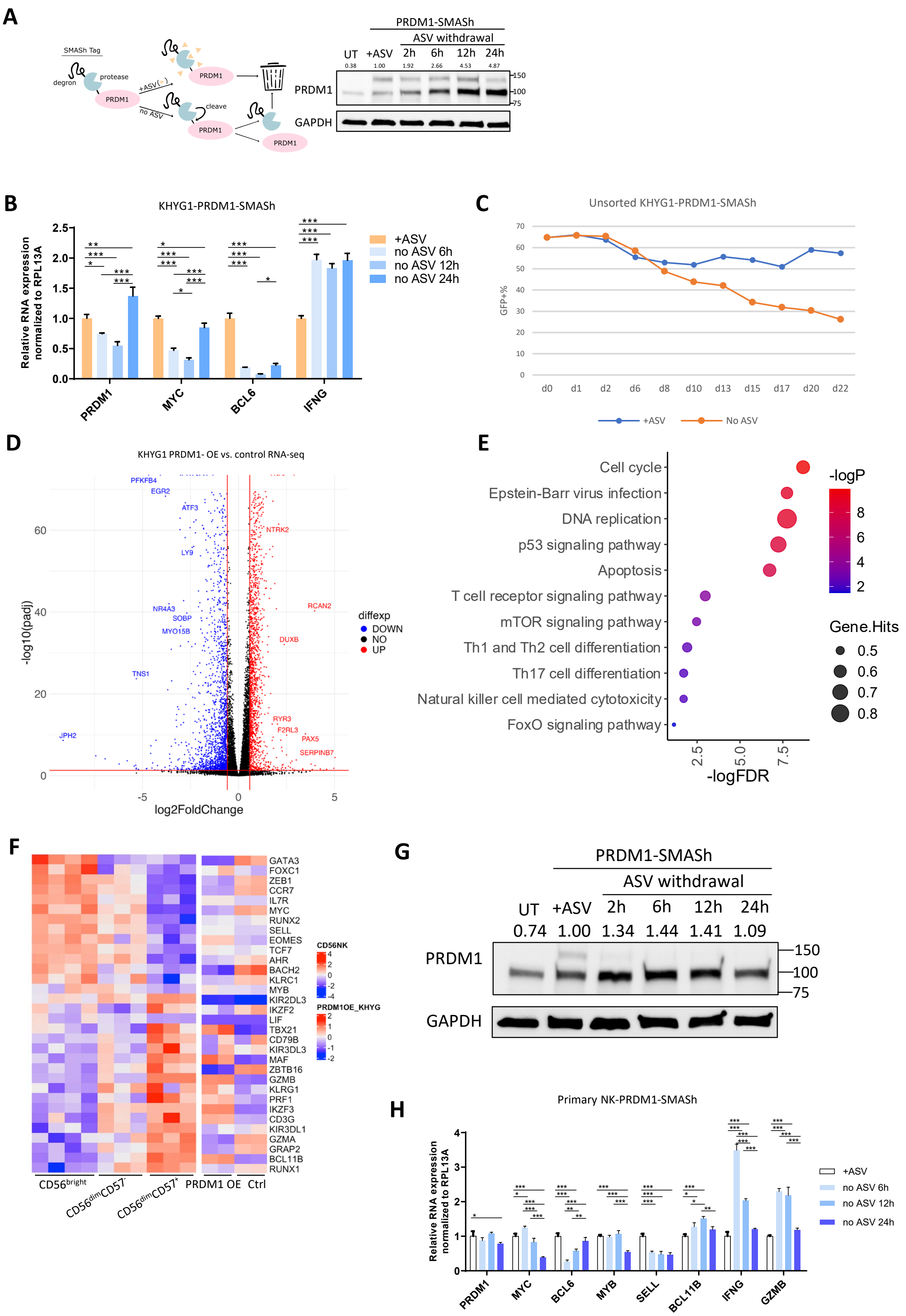

A
SMASh Tag
degron
protease
PRDM1
+ASV
no ASV
cleave
PRDM1-SMASh
ASV withdrawal
UT
+ASV
2h
6h
12h
24h
0.38
1.00
1.92
2.66
4.53
4.87
PRDM1
GAPDH
150
100
75
B
KHYG1-PRDM1-SMASh
Relative RNA expression normalized to RPL13A
+ASV
no ASV 6h
no ASV 12h
no ASV 24h
PRDM1
MYC
BCL6
IFNG
C
Unsorted KHYG1-PRDM1-SMASh
GFP+%
d0
d1
d2
d6
d8
d10
d13
d15
d17
d20
d22
+ASV
No ASV
D
KHYG1 PRDM1- OE vs. control RNA-seq
-log10(padj)
log2FoldChange
PFKFB4
EGR2
ATF3
LY9
NR4A3
SOBP
MYO15B
TNS1
JPH2
NTRK2
RCAN2
DUXB
RYR3
F2RL3
PAX5
SERPINB7
diffexp
DOWN
NO
UP
E
Cell cycle
Epstein-Barr virus infection
DNA replication
p53 signaling pathway
Apoptosis
T cell receptor signaling pathway
mTOR signaling pathway
Th1 and Th2 cell differentiation
Th17 cell differentiation
Natural killer cell mediated cytotoxicity
FoxO signaling pathway
-logFDR
-logP
Gene.Hits
F
GATA3
FOXC1
ZEB1
CCR7
IL7R
MYC
RUNX2
SELL
EOMES
TCF7
AHR
BACH2
KLRC1
MYB
KIR2DL3
IKZF2
LIF
TBX21
CD79B
KIR3DL3
MAF
ZBTB16
GZMB
KLRG1
PRF1
IKZF3
CD3G
KIR3DL1
GZMA
GRAP2
BCL11B
RUNX1
CD56NK
PRDM1OE_KHYG
CD56bright
CD56dimCD57-
CD56dimCD57+
PRDM1 OE
Ctrl
G
PRDM1-SMASh
ASV withdrawal
UT
+ASV
2h
6h
12h
24h
0.74
1.00
1.34
1.44
1.41
1.09
PRDM1
GAPDH
150
100
75
H
Primary NK-PRDM1-SMASh
Relative RNA expression normalized to RPL13A
+ASV
no ASV 6h
no ASV 12h
no ASV 24h
PRDM1
MYC
BCL6
MYB
SELL
BCL11B
IFNG
GZMB

Figure S3

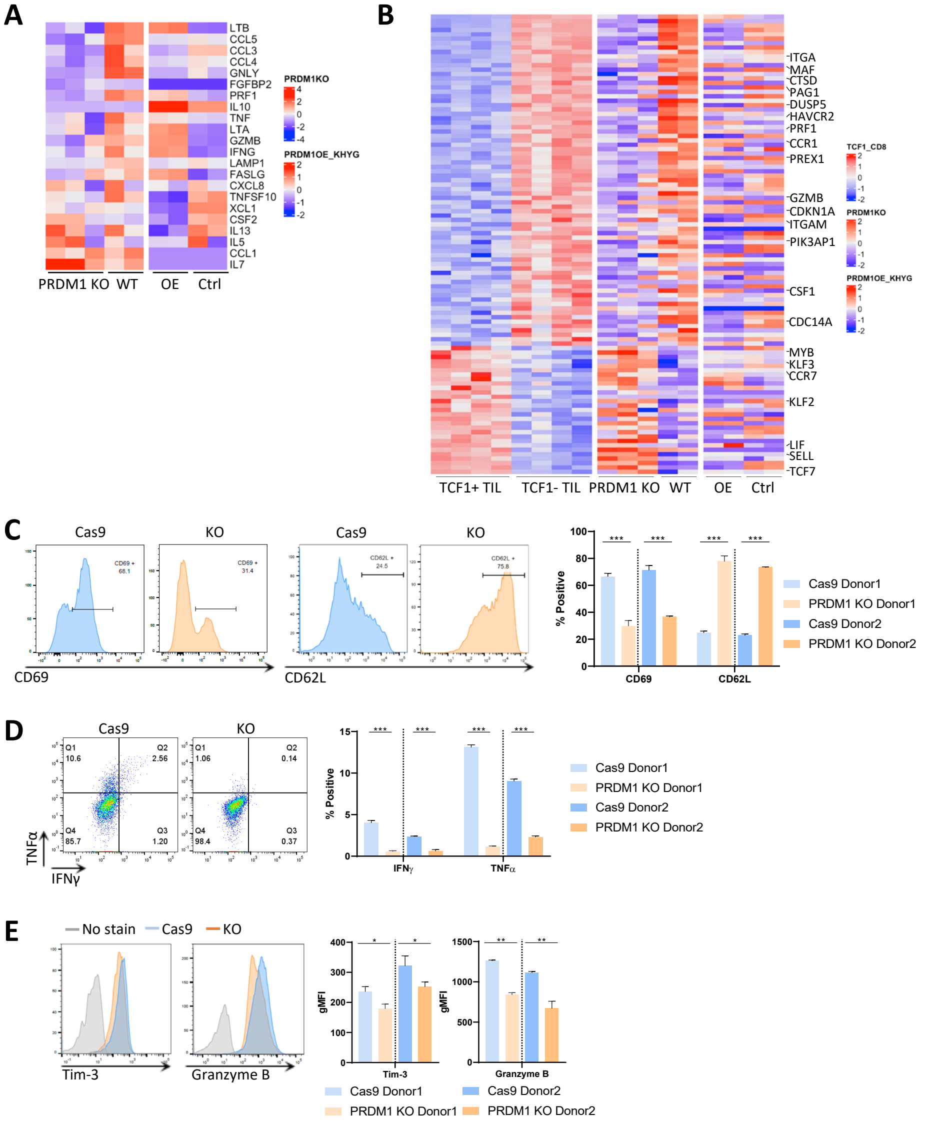

A
LTB
CCL5
CCL3
CCL4
GNLY
FGFBP2
PRF1
IL10
TNF
LTA
GZMB
IFNG
LAMP1
FASLG
CXCL8
TNFSF10
XCL1
CSF2
IL13
IL5
CCL1
IL7
PRDM1KO
PRDM1OE_KHYG
PRDM1 KO
WT
OE
Ctrl
B
ITGA
MAF
CTSD
PAG1
DUSP5
HAVCR2
PRF1
CCR1
PREX1
GZMB
CDKN1A
ITGAM
PIK3AP1
CSF1
CDC14A
MYB
KLF3
CCR7
KLF2
LIF
SELL
TCF7
TCF1_CD8
PRDM1KO
PRDM1OE_KHYG
TCF1+ TIL
TCF1- TIL
PRDM1 KO
WT
OE
Ctrl
C
Cas9
KO
CD69
CD62L
% Positive
Cas9 Donor1
PRDM1 KO Donor1
Cas9 Donor2
PRDM1 KO Donor2
D
Cas9
KO
Q1 10.6
Q2 2.56
Q4 85.7
Q3 1.20
Q1 1.06
Q2 0.14
Q4 98.4
Q3 0.37
TNFα
IFNγ
% Positive
Cas9 Donor1
PRDM1 KO Donor1
Cas9 Donor2
PRDM1 KO Donor2
E
No stain
Cas9
KO
Tim-3
Granzyme B
gMFI
Cas9 Donor1
Cas9 Donor2
PRDM1 KO Donor1
PRDM1 KO Donor2

Figure S4

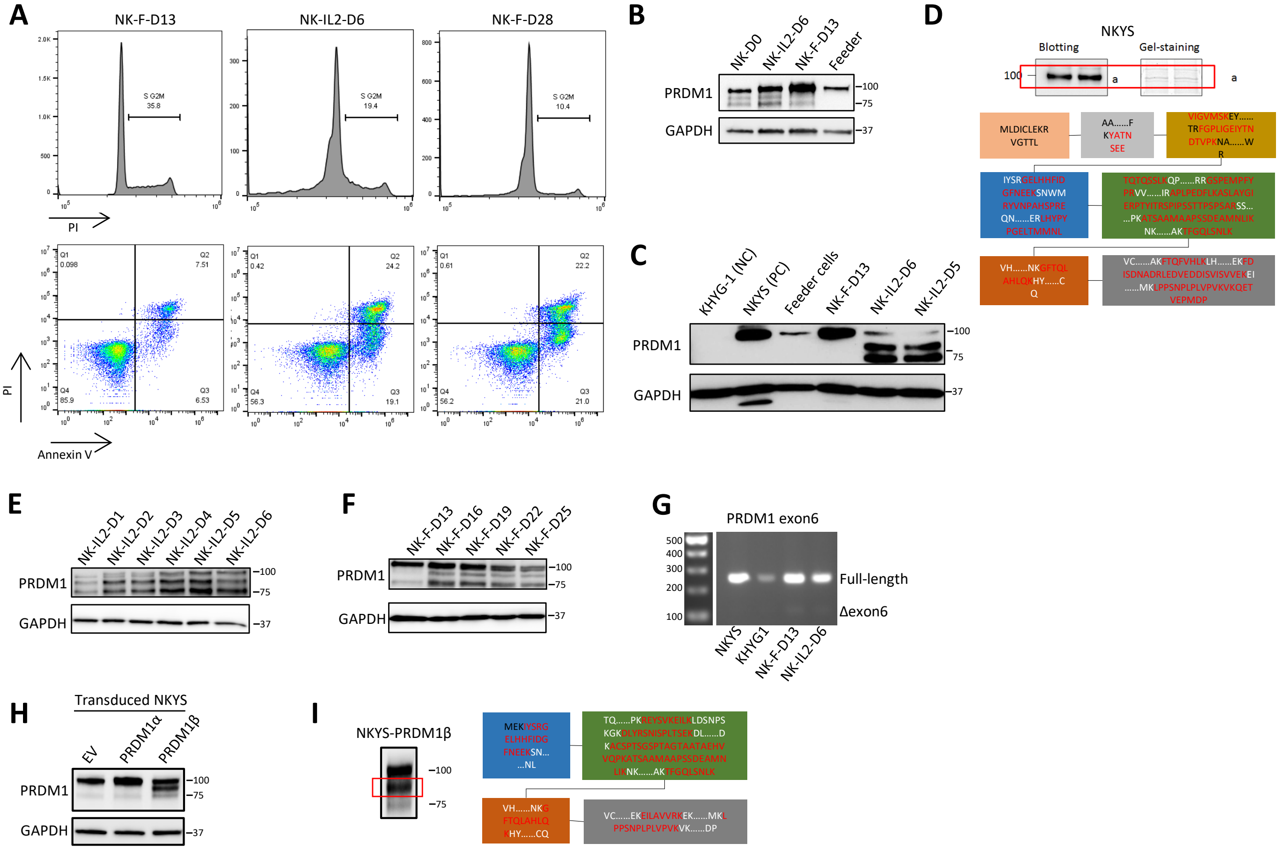

A
NK-F-D13
NK-IL2-D6
NK-F-D28
PI
Annexin V
B
NK-D0
NK-IL2-D6
NK-F-D13
Feeder
PRDM1
GAPDH
C
KHYG-1 (NC)
NKYS (PC)
Feeder cells
NK-F-D13
NK-IL2-D6
NK-IL2-D5
PRDM1
GAPDH
D
NKYS
Blotting
Gel-staining
E
NK-IL2-D1
NK-IL2-D2
NK-IL2-D3
NK-IL2-D4
NK-IL2-D5
NK-IL2-D6
PRDM1
GAPDH
F
NK-F-D13
NK-F-D16
NK-F-D19
NK-F-D22
NK-F-D25
PRDM1
GAPDH
G
PRDM1 exon6
Full-length
Δexon6
NKYS
KHYG1
NK-F-D13
NK-IL2-D6
H
Transduced NKYS
EV
PRDM1α
PRDM1β
PRDM1
GAPDH
I
NKYS-PRDM1β

Figure S5

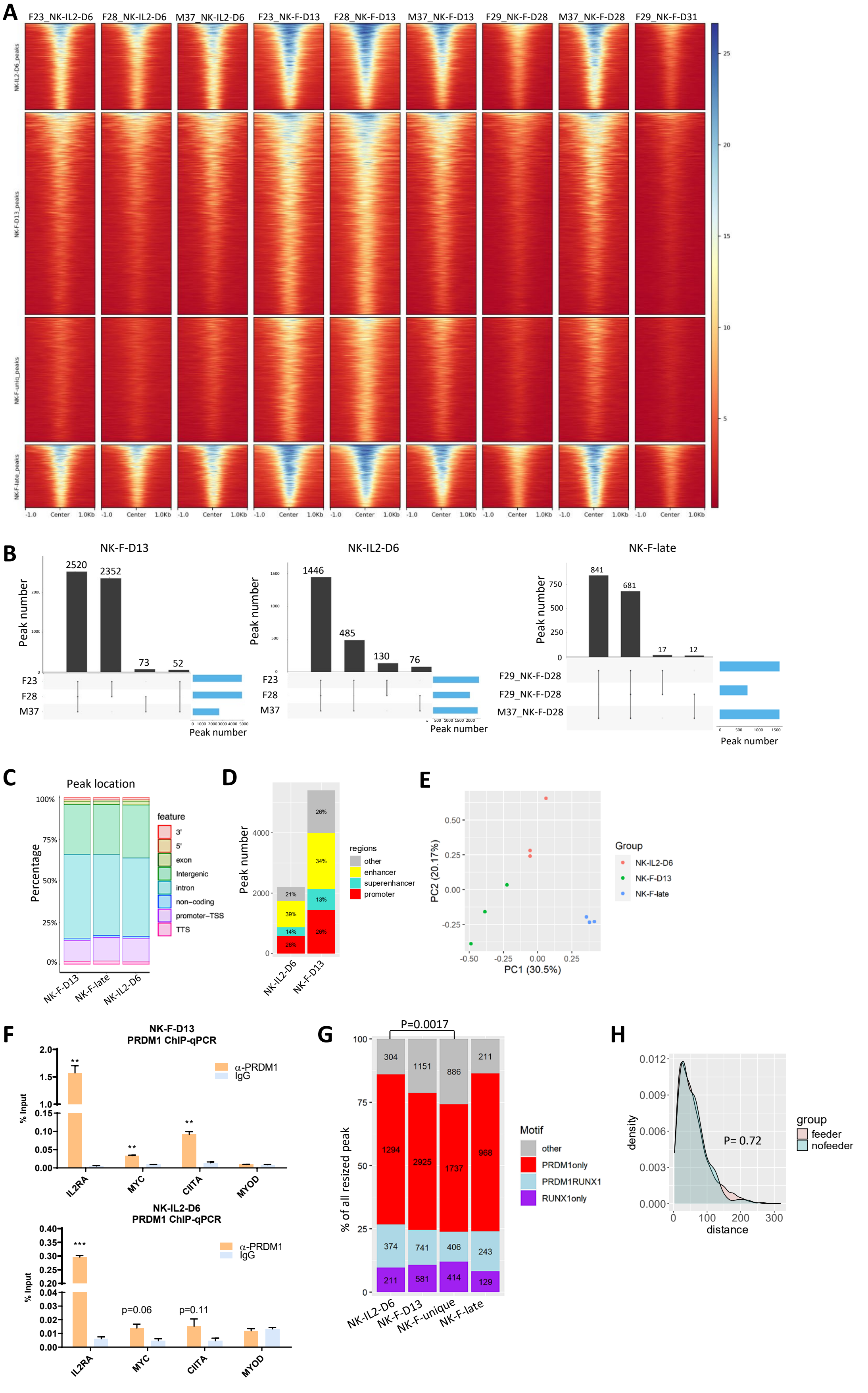
A
F23_NK-IL2-D6
F28_NK-IL2-D6
M37_NK-IL2-D6
F23_NK-F-D13
F28_NK-F-D13
M37_NK-F-D13
F29_NK-F-D28
M37_NK-F-D28
F29_NK-F-D31
NK-IL2-D6_peaks
NK-F-D13_peaks
NK-F-uniq_peaks
NK-F-late_peaks
-1.0 Center 1.0Kb
25
20
15
10
5
B
NK-F-D13
2520
2352
73
52
Peak number
F23
F28
M37
NK-IL2-D6
1446
485
130
76
NK-F-late
841
681
17
12
750
500
250
0
F29_NK-F-D28
F29_NK-F-D28
M37_NK-F-D28
C
Peak location
100%
75%
50%
25%
0%
Percentage
feature
3'
5'
exon
Intergenic
intron
non-coding
promoter-TSS
TTS
NK-F-D13
NK-F-late
NK-IL2-D6
D
Peak number
4000
2000
0
21%
39%
14%
25%
26%
34%
13%
26%
regions
other
enhancer
superenhancer
promoter
NK-IL2-D6
NK-F-D13
E
PC2 (20.17%)
0.50
0.25
0.00
-0.25
-0.50
0.00
0.25
PC1 (30.5%)
Group
NK-IL2-D6
NK-F-D13
NK-F-late
F
NK-F-D13
PRDM1 ChIP-qPCR
% Input
α-PRDM1
IgG
IL2RA
MYC
CIITA
MYOD
NK-IL2-D6
PRDM1 ChIP-qPCR
p=0.06
p=0.11
G
P=0.0017
% of all resized peak
100
75
50
25
0
304
1151
886
211
1294
2925
1737
968
374
741
406
243
211
581
414
129
Motif
other
PRDM1only
PRDM1RUNX1
RUNX1only
NK-IL2-D6
NK-F-D13
NK-F-unique
NK-F-late
H
density
0.012
0.009
0.006
0.003
0.000
P= 0.72
0
100
200
300
distance
group
feeder
nofeeder

Figure S6

A NK-IL2-D6

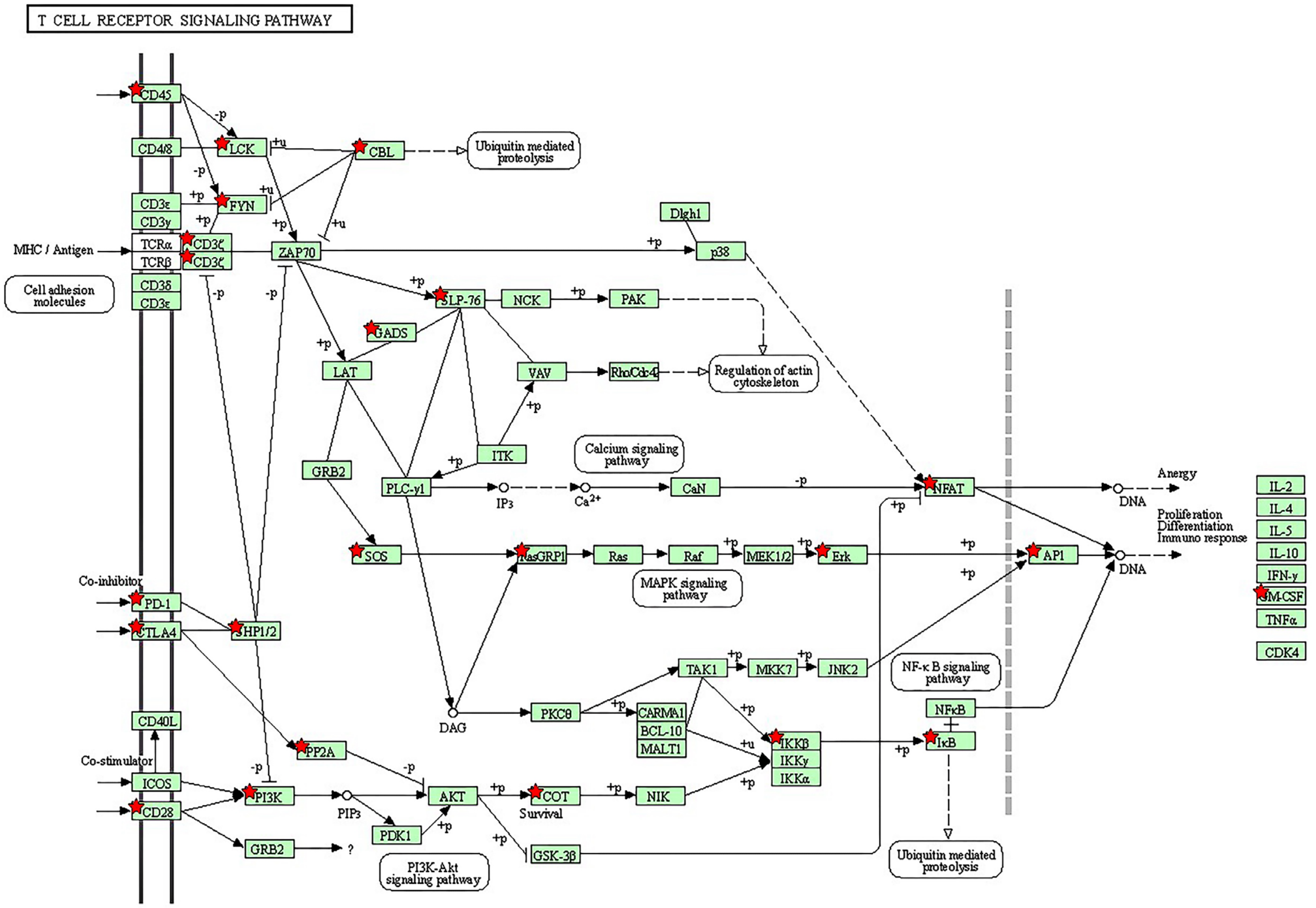

B NK-F-D13

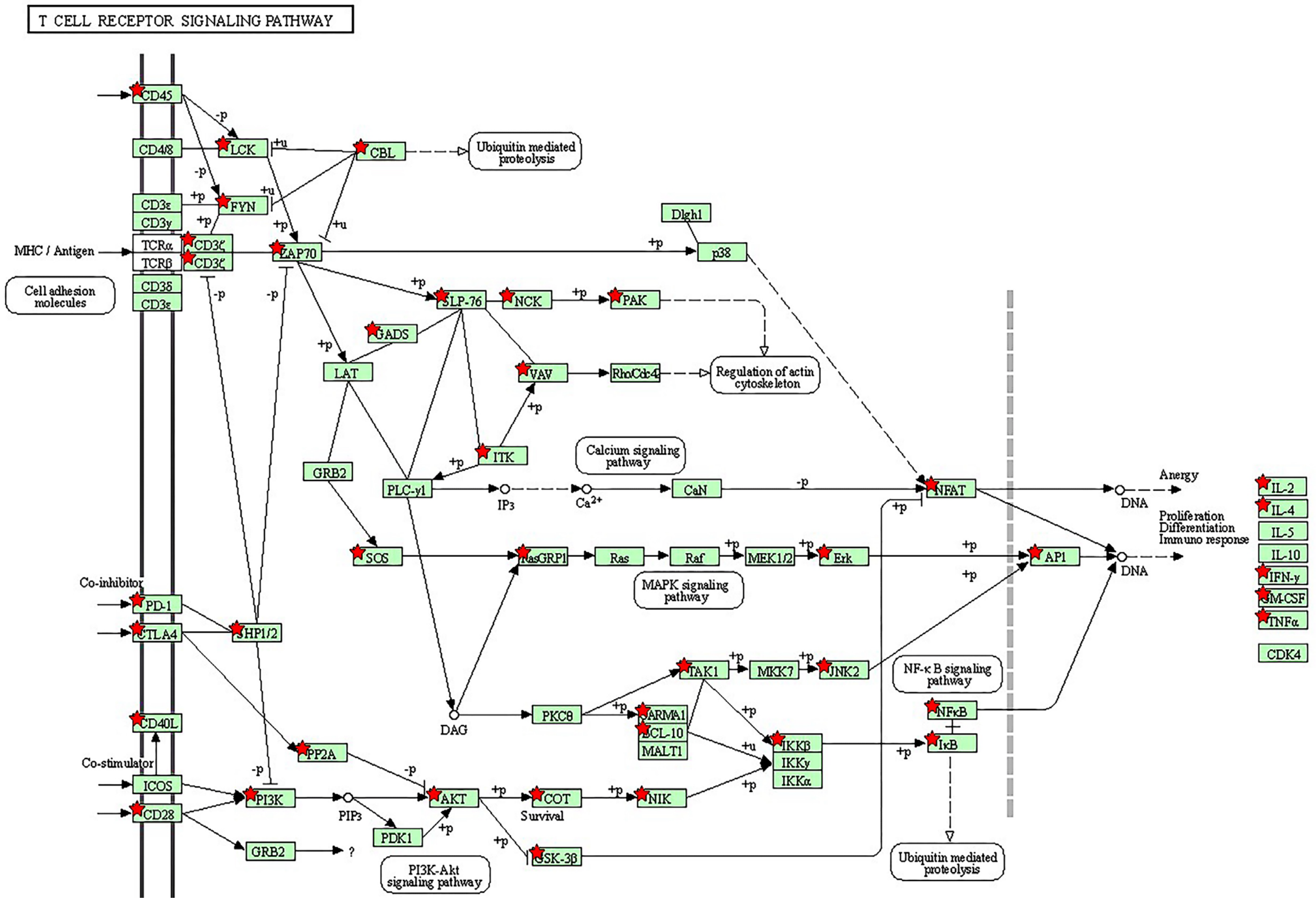

Figure S6, cont.

C NK-IL2-D6

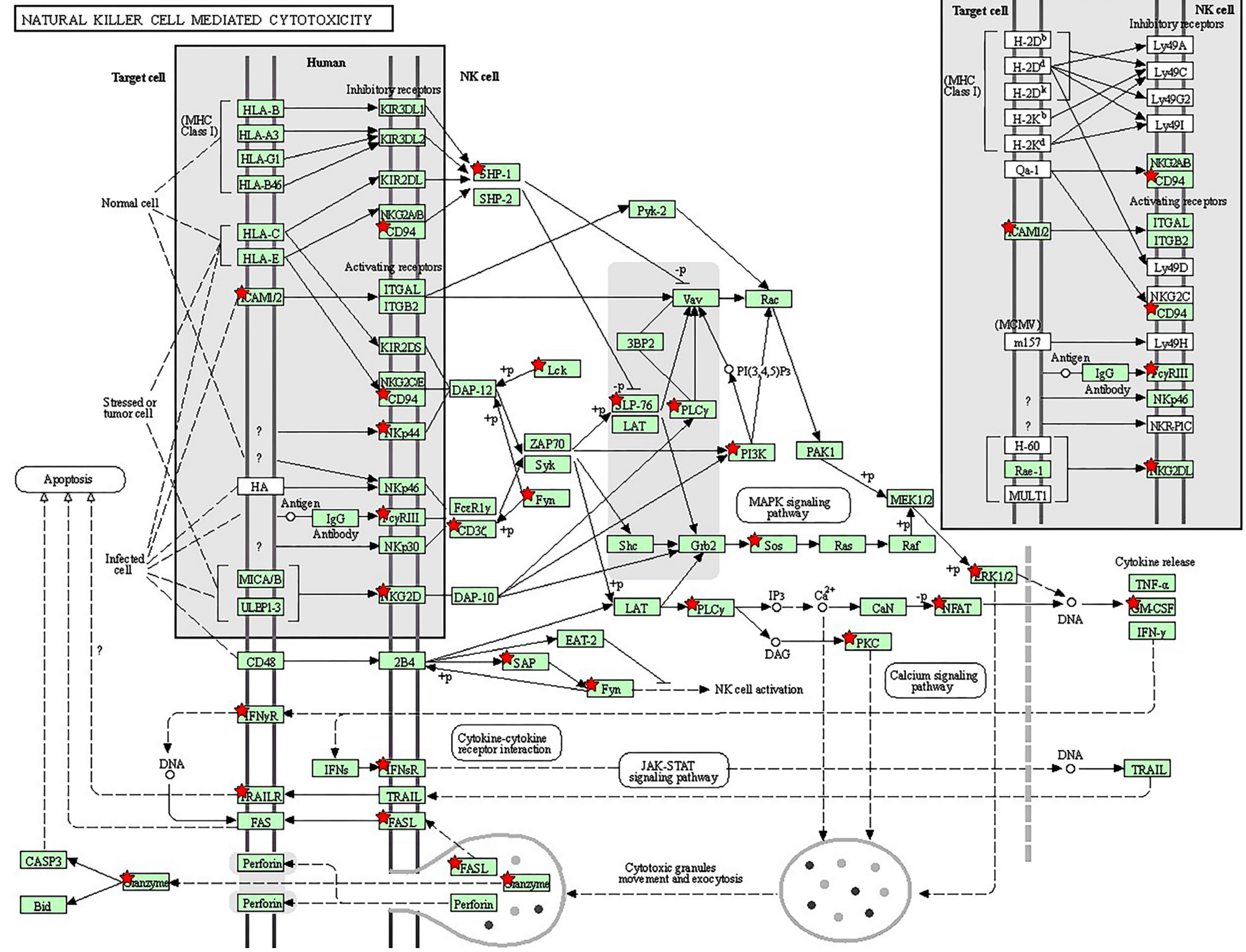

D NK-F-D13

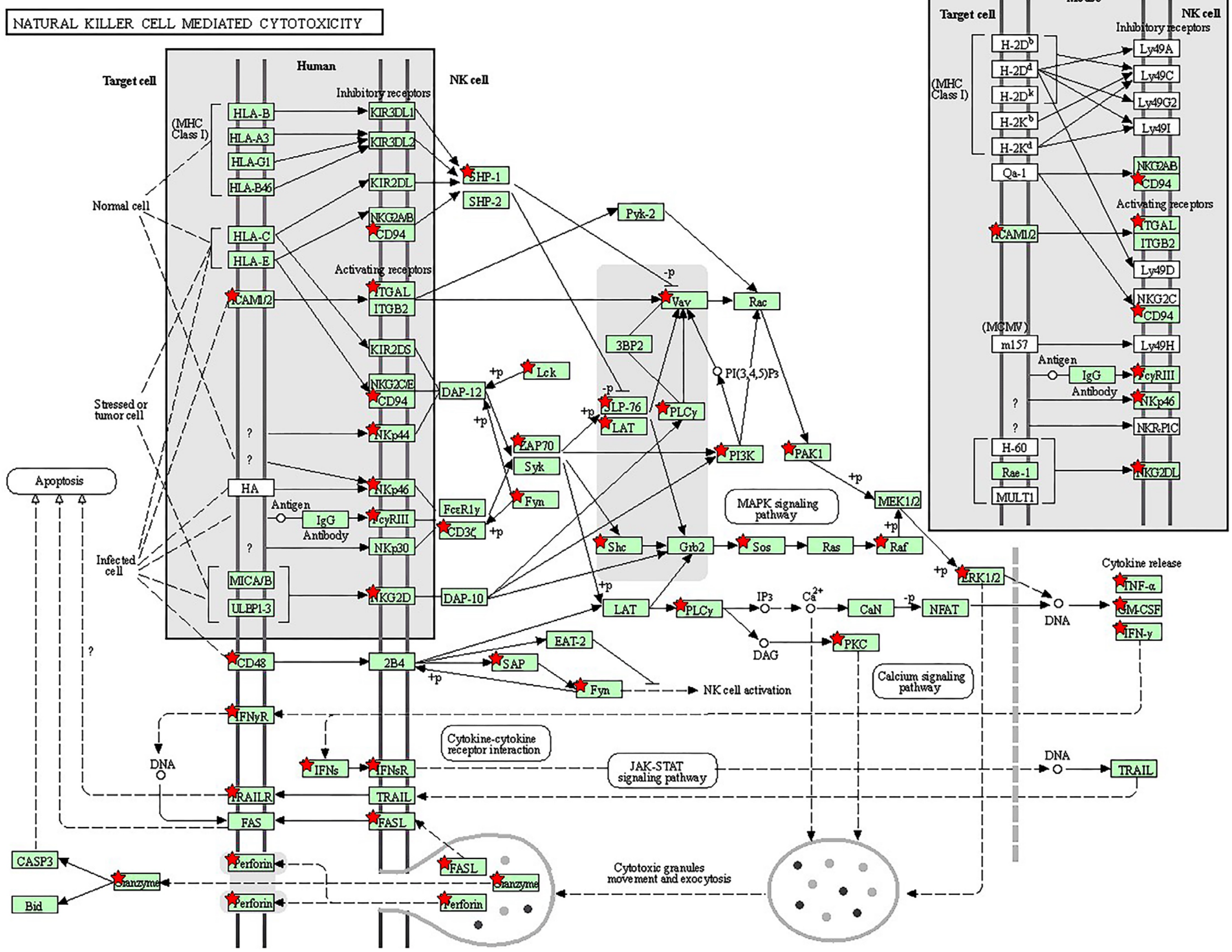

Figure S7

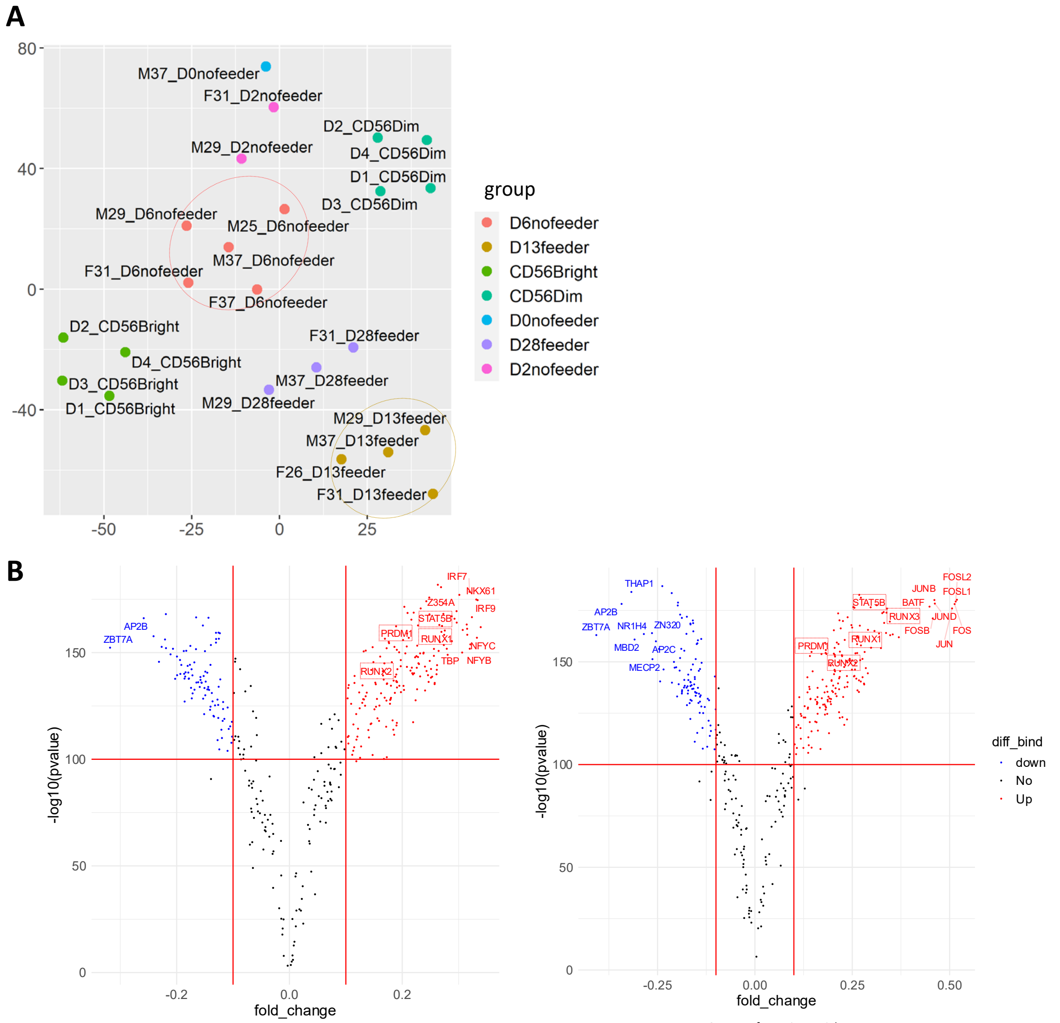

ATAC-seq TF footprint enrichment:
NK-IL2-d6 vs freshly isolated CD56^{dim} NK-cells

ATAC-seq TF footprint enrichment:
NK-F-D13 vs freshly isolated CD56^{dim} NK-cells

Figure S8

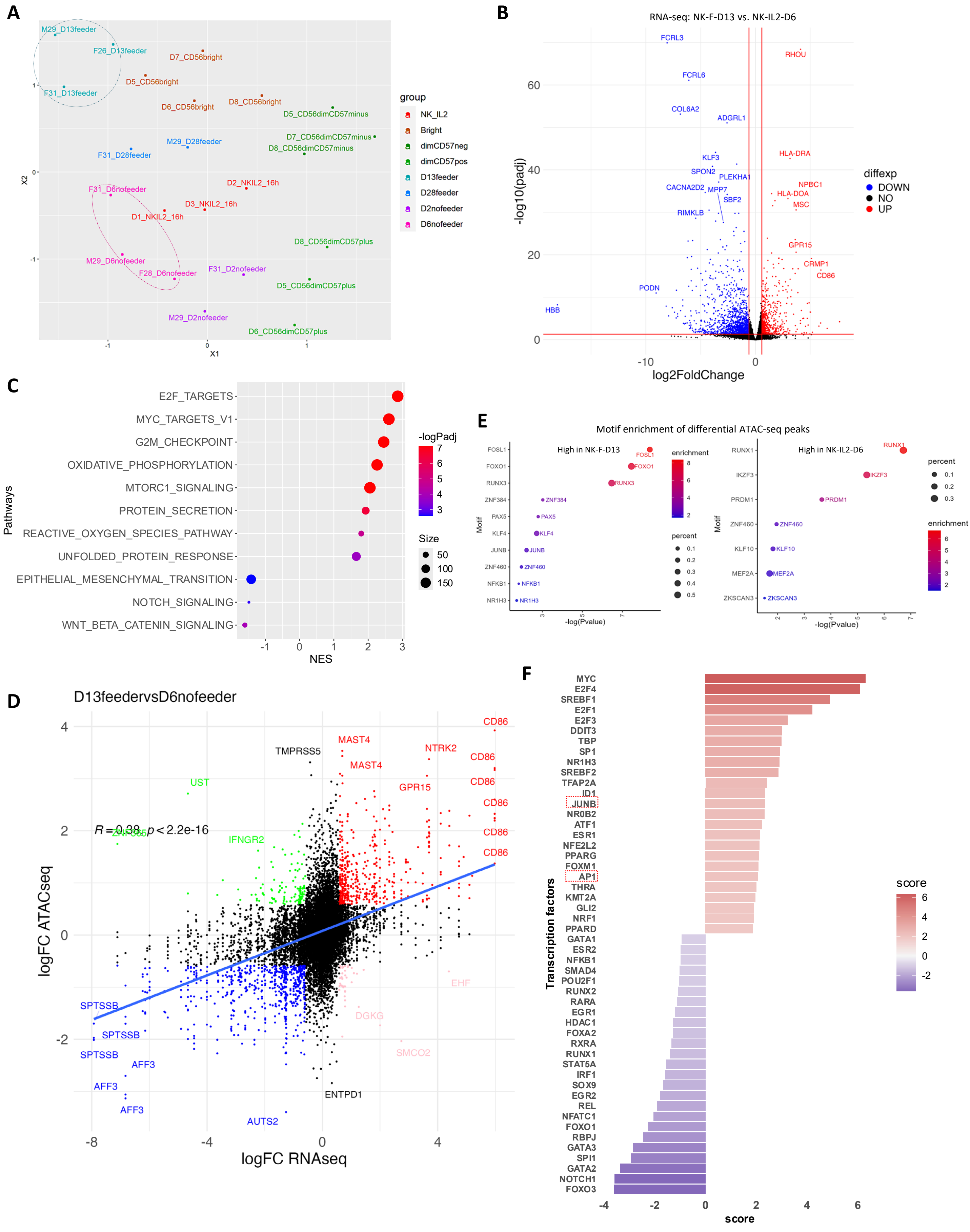
A
M29_D13feeder
F26_D13feeder
F31_D13feeder
D7_CD56bright
D5_CD56bright
D6_CD56bright
D8_CD56bright
D5_CD56dimCD57minus
D7_CD56dimCD57minus
D8_CD56dimCD57minus
M29_D28feeder
F31_D28feeder
F31_D6nofeeder
D2_NKIL2_16h
D3_NKIL2_16h
D1_NKIL2_16h
D8_CD56dimCD57plus
M29_D6nofeeder
F28_D6nofeeder
F31_D2nofeeder
D5_CD56dimCD57plus
M29_D2nofeeder
D6_CD56dimCD57plus
X1
X2
group
NK_IL2
Bright
dimCD57neg
dimCD57pos
D13feeder
D28feeder
D2nofeeder
D6nofeeder
B
RNA-seq: NK-F-D13 vs. NK-IL2-D6
FCRL3
RHOU
FCRL6
COL6A2
ADGRL1
KLF3
HLA-DRA
SPON2
PLEKHA1
CACNA2D2
MPP7
NPBC1
HLA-DOA
SBF2
MSC
RIMKLB
GPR15
CRMP1
CD86
PODN
HBB
-log10(padj)
log2FoldChange
diffexp
DOWN
NO
UP
C
E2F_TARGETS
MYC_TARGETS_V1
G2M_CHECKPOINT
OXIDATIVE_PHOSPHORYLATION
MTORC1_SIGNALING
PROTEIN_SECRETION
REACTIVE_OXYGEN_SPECIES_PATHWAY
UNFOLDED_PROTEIN_RESPONSE
EPITHELIAL_MESENCHYMAL_TRANSITION
NOTCH_SIGNALING
WNT_BETA_CATENIN_SIGNALING
Pathways
NES
-logPadj
Size
50
100
150
E
Motif enrichment of differential ATAC-seq peaks
High in NK-F-D13
FOSL1
FOXO1
RUNX3
ZNF384
PAX5
KLF4
JUNB
ZNF460
NFKB1
NR1H3
Motif
-log(Pvalue)
enrichment
percent
High in NK-IL2-D6
RUNX1
IKZF3
PRDM1
ZNF460
KLF10
MEF2A
ZKSCAN3
D
D13feedervsD6nofeeder
R = 0.38, p < 2.2e-16
CD86
MAST4
NTRK2
TMPRSS5
GPR15
UST
IFNGR2
EHF
DGKG
SMCO2
SPTSSB
AFF3
AUTS2
ENTPD1
logFC ATACseq
logFC RNAseq
F
MYC
E2F4
SREBF1
E2F1
E2F3
DDIT3
TBP
SP1
NR1H3
SREBF2
TFAP2A
ID1
JUNB
NR0B2
ATF1
ESR1
NFE2L2
PPARG
FOXM1
AP1
THRA
KMT2A
GLI2
NRF1
PPARD
GATA1
ESR2
NFKB1
SMAD4
POU2F1
RUNX2
RARA
EGR1
HDAC1
FOXA2
RXRA
RUNX1
STAT5A
IRF1
SOX9
EGR2
REL
NFATC1
FOXO1
RBPJ
GATA3
SPI1
GATA2
NOTCH1
FOXO3
Transcription factors
score

Figure S9

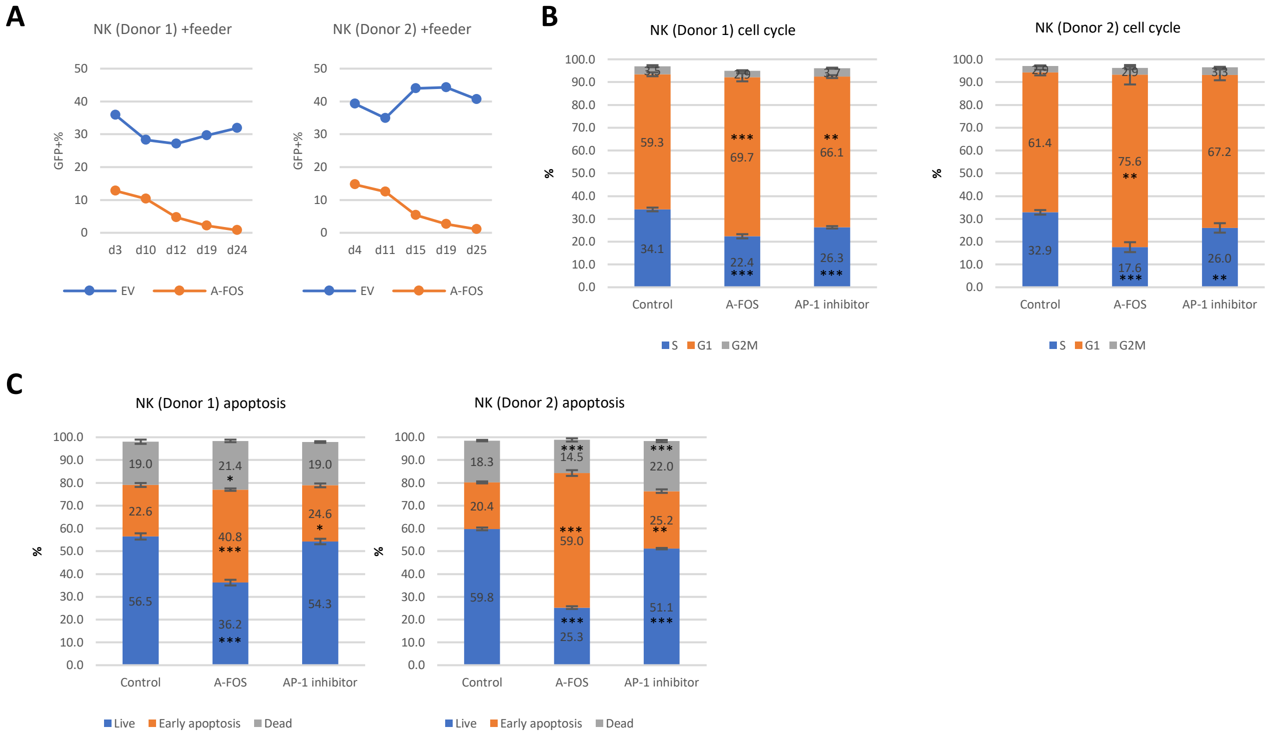
A
NK (Donor 1) +feeder
NK (Donor 2) +feeder
GFP+%
d3 d10 d12 d19 d24
d4 d11 d15 d19 d25
EV
A-FOS
B
NK (Donor 1) cell cycle
NK (Donor 2) cell cycle
%
Control
A-FOS
AP-1 inhibitor
S
G1
G2M
59.3
69.7
66.1
34.1
22.4
26.3
61.4
75.6
67.2
32.9
17.6
26.0
C
NK (Donor 1) apoptosis
NK (Donor 2) apoptosis
Live
Early apoptosis
Dead
19.0
21.4
19.0
22.6
40.8
24.6
56.5
36.2
54.3
18.3
14.5
22.0
20.4
59.0
25.2
59.8
25.3
51.1

Figure S10

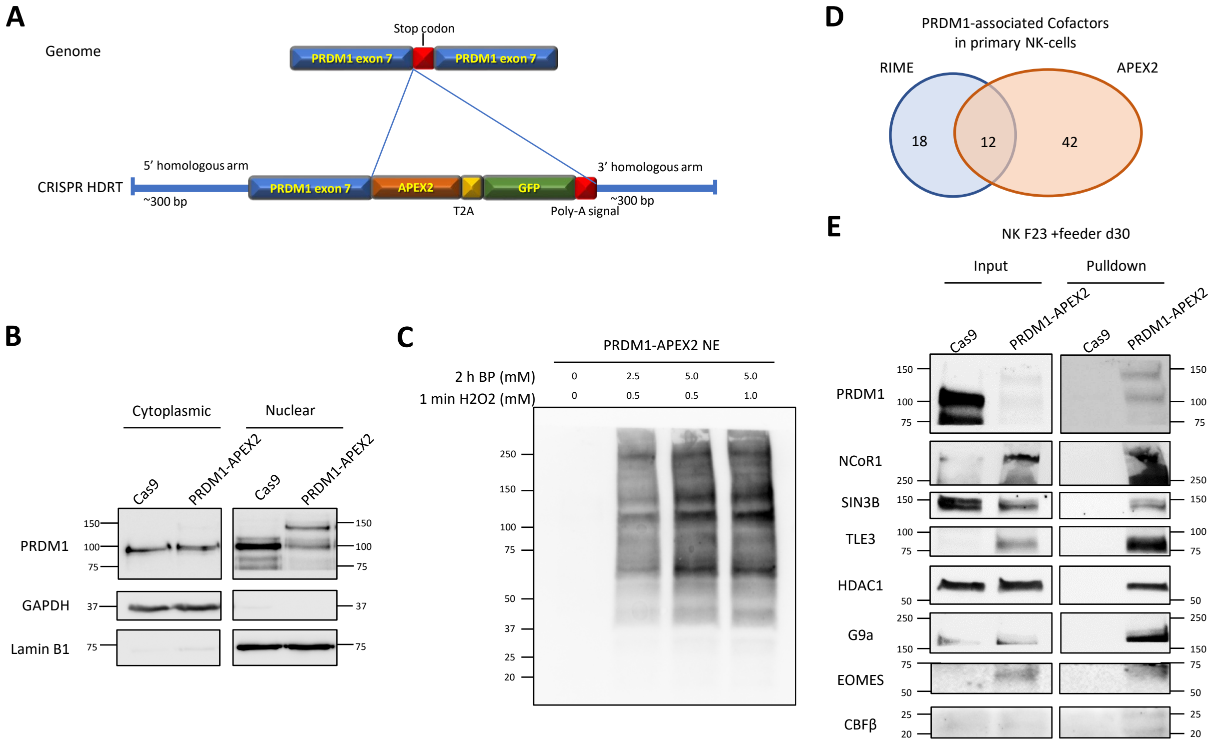

A
Genome
Stop codon
PRDM1 exon 7
PRDM1 exon 7
CRISPR HDRT
5' homologous arm
~300 bp
PRDM1 exon 7
APEX2
T2A
GFP
Poly-A signal
3' homologous arm
~300 bp
B
Cytoplasmic
Nuclear
Cas9
PRDM1-APEX2
Cas9
PRDM1-APEX2
PRDM1
GAPDH
Lamin B1
C
PRDM1-APEX2 NE
2 h BP (mM) 0 2.5 5.0 5.0
1 min H2O2 (mM) 0 0.5 0.5 1.0
D
PRDM1-associated Cofactors
in primary NK-cells
RIME
APEX2
18
12
42
E
NK F23 +feeder d30
Input
Pulldown
Cas9
PRDM1-APEX2
Cas9
PRDM1-APEX2
PRDM1
NCoR1
SIN3B
TLE3
HDAC1
G9a
EOMES
CBFβ
