# Supplementary material for: Key regulatory roles of PRDM1 in human NK-cell differentiation and activation
Source: Leukemia. 2025 Dec 9;40(1):199–210. doi: 10.1038/s41375-025-02815-z (PMC12789010; doi:10.1038/s41375-025-02815-z)
Supplement: Supplementary file 2 — Supplementary Figure and Table Legend [file 41375_2025_2815_MOESM2_ESM.docx]

1. **Supplementary Figure Legend**
2. **Figure S1. *PRDM1* functional knockout in primary NK-cells.** **A.** Schematic overview of human NK-cells cultured in IL-2 with or without feeder cells. **B**. Enrichment of CD56^+^ NK-cells in freshly isolated NK-cells, NK-IL2-D6, and NK-F-D13. CD235a was used as a marker for the feeder cells and indicated that there were no feeder cells in NK-F-D13. **C**. Genotyping of *PRDM1* fKO by PCR. *PRDM1*-fKO cells showed smaller MW of DNA fragments that amplifies the sgRNA targeted exon 4 of PRDM1 (top panel), due to fragment deletion indicated by ICE analysis (bottom panel). **D**. GSEA graphs showing similar GEP between *PRDM1*-fKO NK-cells with less differentiated CD56^bright^ cells and TCF1^+^ TILs. **E**. Flow cytometric analysis of CD16 expression measured by percentage of positive cells in *PRDM1*-fKO and control NK-cells. **F**. qRT-PCR of PRDM1 target genes in *PRDM1*-fKO NK-cells compared to WT cells. *n* = 3, two independent experiments, two-tailed Student’s t test; *, p < 0.05; **, p < 0.01; ***, p < 0.001.
3. **Figure S2. *PRDM1* re-expression systems used in the KHYG1 cell line. A**. Schematic overview of the SMASh-tagged PRDM1 inducible system controlled by the protease inhibitor ASV (left) and Western blotting of PRDM1 overexpression in KHYG1 cells using the SMASh system (right). Expression levels of PRDM1α normalized to GAPDH levels were indicated above each lane. **B**. qRT-PCR of selected PRDM1 target genes in KHYG1 cells with (no ASV) or without (+ASV) *PRDM1* OE at indicated time points. **C**. Growth of KHYG1 cells with *PRDM1* OE monitored by the percentage of GFP^+^ cells. **D.** Volcano plot showing DEGs (fold change > 1.5, padj < 0.05) between PRDM1 OE and control KHYG1 cells. **E**. Pathway analysis of DEGs between *PRDM1*-OE and control KHYG1 cells. **F**. Heatmaps showing selected DEGs between *PRDM1* OE vs. control KHYG1 that were associated with human NK-cell differentiation from CD56^bright^, CD56^dim^CD57^-^, to CD56^dim^CD57^+^ cells. **G**. Western blotting of PRDM1 overexpression in primary NK-cells using the SMASh system. Expression levels of PRDM1α normalized to GAPDH levels were indicated above each lane. **H**. qRT-PCR of selected PRDM1 target genes in primary NK-cells with or without *PRDM1* OE at indicated time points. *n* = 3, two independent experiments, two-tailed Student’s t test; For panels f and k: *n* = 3, two independent experiments, one-way ANOVA; *, p < 0.05; **, p < 0.01; ***, p < 0.001.
4. **Figure S3. *PRDM1*-fKO NK cells showed a less mature phenotype. A**. Heatmap showing the expression of key genes related to NK-cell effector function in *PRDM1*-fKO vs. WT NK-cells and PRDM1 OE vs. control KHYG1 cells. **B**. Heatmaps showing selected DEGs between *PRDM1*-fKO vs. WT primary human NK-cells or PRDM1 OE vs. control KHYG1 that were associated with the differences between TCF1^+^ progenitor exhausted tumor infiltrating lymphocytes (TILs) and TCF1^-^ terminally exhausted TILs. **C-E**. Flow cytometric analysis of CD69 (C), CD62L (C), IFNγ (D), TNFα (D) measured by percentage of positive cells and Tim-3 (E), granzyme B (E) measured by geometric mean fluorescent intensity (gMFI) in *PRDM1*-fKO NK cells and WT cells.
5. **Figure S4. Different isoforms of PRDM1 were expressed in human NK-cells stimulated with feeder cells or IL-2 alone.** **A**. Flow cytometric analysis of proliferation and apoptosis in NK-F-D13, NK-IL2-D6, and NK-F-D28. **B-C**. Western blotting of PRDM1 isoforms in non-stimulated (NK-D0, freshly isolated) or IL-2/feeder cell stimulated primary human NK-cells (B) or in stimulated NK cells with KHYG-1 as a negative control and NKYS as a positive control for PRDM1 expression (C). NK-cells stimulated with feeder cells for 13 days were denoted as NK-F-D13, while NK-cells cultured with IL-2 alone for 6 days were marked as NK-IL2-D6. **D**. Mass spectrometric analysis of PRDM1 top band from NKYS cells corresponding to PRDM1α sequence. Each box shows peptides encoded by one exon, and peptide sequences detected in mass spectrometric analysis were highlighted in red. MS detected peptides covering all PRDM1 exons except exon 1, which was likely due to the short amino acid sequence of exon 1. **E-F**. Western blotting of PRDM1 isoforms in primary human NK-cells cultured with IL-2 alone (E) or with feeder cell stimulation (F) at different time points. **G**. Agarose gel showing very low level of PCR amplification of the ∆exon6 template. **H**. Western blotting of PRDM1 in NKYS cells transduced with PRDM1α or PRDM1β. EV, empty vector. **I**. Mass spectrometric analysis of PRDM1 middle band from NKYS cells transduced with PRDM1β coding sequence, showing peptides sequences corresponding to PRDM1β.
6. **Figure S5. Global analysis of PRDM1-bound sites in human NK-cells.** **A**. Heatmap of PRDM1 ChIP-seq peaks at different regions in individual samples. **B**. UpSet plots showing the number of consensus peaks present in any two of the three replicate samples in NK-F-D13, NK-IL2-D6, and NK-F-late. These consensus peaks were chosen for subsequent analyses. **C**. Bar graph showing the percentage of peaks found in indicated regions. **D**. Bar graph showing the number of PRDM1 ChIP-seq peaks located in promoters (TSS+-1kb) annotated by Homer or in enhancers identified in H3K27ac sequencing data by Homer. **E**. PCA of PRDM1 ChIP-seq samples based on the number of mapped reads at each peak. **F**. PRDM1 ChIP-qPCR analysis of known PRDM1 target genes and control gene *MYOD* in NK-IL2-D6 and NK-F-D13. *n* = 2, two independent experiments, two-tailed Student’s t test; *, p < 0.05; **, p < 0.01; ***, p < 0.001. **G**. Bar graph showing percentage of resized peaks (summits ± 200bp) containing PRDM1 only, RUNX only, both PRDM1 and RUNX motifs, or other motifs. P-value indicates the significance of Chi-squared test of percentage between two groups. **H**. Distance between PRDM1 and RUNX1 motifs within PRDM1 ChIP peaks that contain both motifs. P-value denotes the significance of Kolmogorov–Smirnov test of the two distributions.
7. **Figure S6. Pathway analysis of PRDM1 bound genes. A-B**. KEGG pathways showing PRDM1-bound genes (marked with stars) in T cell receptor signaling pathway in NK-IL2-D6 (A) and NK-F-D13 (B). **C-D**. KEGG pathways showing PRDM1-bound genes (marked with stars) in NK-cell mediated cytotoxicity in NK-IL2-D6 (C) and NK-F-D13 (D).
8. **Figure S7. ATAC-seq analysis of stimulated NK-cells.** **A**. T-SNE plot of ATAC-seq showing clustering of NK-cells cultured in different conditions as well as freshly isolated CD56^dim^ and CD56^bright^ NK-cells (GSE112813). **B**, Volcano plots showing significantly enriched TF footprints in ATAC-seq comparing IL-2 (left) or feeder (right) stimulated NK-cells with resting CD56^dim^ NK-cells.
9. **Figure S8**. **RNA-seq analysis of stimulated NK-cells.** **A**. UMAP plot of RNA-seq showing clustering of NK-cells cultured in different conditions and NK-cells from GSE112813 and GSE130286. **B**. Volcano plot showing DEGs (fold change > 1.5, padj < 0.05) between NK-F-D13 and NK-IL2-D6. **C**. Pathway analysis of DEGs comparing NK-F-D13 and NK-IL2-D6. **D**. Scatter plot showing the positive correlation of PRDM1-bound genes between differential chromatin accessibility and RNA expression comparing NK-F-D13 and NK-IL2-D6. **E**. Motif enrichment of differential ATAC-seq peaks by STREME. P value indicates the possibility of identifying the indicated motifs within the peaks randomly. **F**. Upstream TF regulators predicted by the DEGs between NK-F-D13 and NK-IL2-D6.
10. **Figure S9. AP-1 inhibition represses NK-cell growth with feeder cell stimulation. A**. Tracking of GFP^+^ cell percentage in coculture of A-FOS (in a GFP-expressing construct) or empty vector (EV) transduced and untransduced NK-cells. Feeder cells were added every week. **B-C**. Cell cycle (B) and apoptosis (C) assay of A-FOS transduced or AP-1 inhibitor T5224 treated NK-cells at day 4 after feeder cell stimulation. For panels B and C, *n* = 3, two independent experiments, one-way ANOVA; *, p < 0.05; **, p < 0.01; ***, p < 0.001, indicating the comparison with control.
11. **Figure S10. APEX2 modification of PRDM1, biotinylation, and pulldown.** **A**. CRISPR homology-directed repair template (HDRT) design of PRDM1-APEX2 fusion genomic modification. **B**. Nuclear extraction test. Cytoplasmic and nuclear fraction were collected for Western blotting analysis of cytoplasmic marker GAPDH and nuclear marker Lamin B1. **C**. Biotin labeling test using nuclear extracts. Different concentrations of biotin-phenol (BP) and H_2_O_2_ were tested. 5mM BP 2h and 0.5 mM H_2_O_2_ 1min were used for the subsequent biotin labeling experiments. **D**. Venn diagram showing the number of overlapping PRDM1 associated cofactors identified by RIME and biotinylation experiments. **E**. Western blotting analysis of PRDM1-associated proteins. Unmodified (Cas9) or APEX2-modified primary human NK-cells were collected after feeder stimulation for 30 days, biotin-labeled with the conditions mentioned above. Nuclear extract was prepared, incubated with Streptavidin Sepharose beads for 4 h, and then thoroughly washed before loading for Western blotting analysis.
12. **Supplementary Tables**
13. Supplementary Table 1. RNA-seq comparing DEGs between PRDM1 fKO and WT human NK-cells.
14. Supplementary Table 2. RNA-seq comparing DEGs between PRDM1 OE and control KHYG1 cells.
15. Supplementary Table 3. ChIP-seq consensus peaks in NK-F-D13 annotated by Homer.
16. Supplementary Table 4. ChIP-seq consensus peaks in NK-IL2-D6 annotated by Homer.
17. Supplementary Table 5. ChIP-seq consensus peaks in NK-F-late annotated by Homer.
18. Supplementary Table 6. ATAC-seq comparing differential peaks between NK-F-D13 and freshly isolated CD56dim NK-cells.
19. Supplementary Table 7. ATAC-seq comparing differential peaks between NK-IL2-D6 and freshly isolated CD56dim NK-cells.
20. Supplementary Table 8. ATAC-seq comparing differential peaks between NK-F-D13 and NK-IL2-D6.
21. Supplementary Table 9. ATAC-seq footprint enrichment analysis between NK-F-D13 and freshly isolated CD56dim NK-cells.
22. Supplementary Table 10. ATAC-seq footprint enrichment analysis between NK-IL2-D6 and freshly isolated CD56dim NK-cells.
23. Supplementary Table 11. ATAC-seq footprint enrichment analysis between NK-F-D13 and NK-IL2-D6.
24. Supplementary Table 12. RIME assay identification of PRDM1-interacting peptides.
25. Supplementary Table 13. APEX2 proximity-based biotinylation assay identification of PRDM1-interacting peptides.
26. Supplementary Table 14. KEGG pathway analysis of PRDM1 bound genes.
27. Supplementary Table 15. KEGG pathway analysis of overlapping genes between PRDM1-bound genes and DEGs in RNA-seq.
28. Supplementary Table 16. KEGG pathway analysis of PRDM1 footprint-containing genes that showed differential ATAC signal between NK-IL2-d6 or NK-F-D13 vs. freshly isolated CD56dim NK-cells.
29. Supplementary Table 17. KEGG pathway analysis of all differential ATAC-seq peaks and AP-1 footprint-containing differential ATAC-seq peaks comparing NK-F-D13 and NK-IL2-D6.
30. Supplementary Table 18. KEGG pathway analysis of genes with both AP-1 and PRDM1 footprint that showed higher ATAC-seq signal in NK-F-D13 compared to NK-IL2-D6.
