# Supplementary material for: Key regulatory roles of PRDM1 in human NK-cell differentiation and activation
Source: Leukemia. 2025 Dec 9;40(1):199–210. doi: 10.1038/s41375-025-02815-z (PMC12789010; doi:10.1038/s41375-025-02815-z)
Supplement: Supplementary file 4 — Supplemental Material and Methods [file 41375_2025_2815_MOESM4_ESM.docx]

1. Supplemental Material and Methods

# NK-cell enrichment and culture

1. NK-cells were isolated from peripheral blood mononuclear cells (PBMCs) of healthy donors of random
2. sex/gender by negative selection using EasySep Human NK-cell Enrichment Kit (STEMCELL, USA, #17955)
3. according to the manufacturer’s protocol. The purity of enriched NK-cell samples was all above 95%, as
4. determined by flow cytometric analysis using anti-CD56 PE (Biolegend, USA, #304605) and anti-CD3 FITC
5. (Biolegend, USA, #100305) (Fig. S2a, left panel). The NK-cells were either co-cultured with feeder cells (an
6. engineered feeder cell line, K562-Cl9-mb21^1^, irradiated using 100 Gy prior to coculture) at 1:10 ratio of
7. NK:feeder or cultured without feeder cells. These NK-cells were cultured in X-VIVO 15 Serum-free
8. Hematopoietic Cell Medium (Lonza, #04-418Q, Basel, Switzerland) with 10% FBS (HyClone, USA, #SH30070),
9. 1% (v/v) penicillin-streptomycin solution (Invitrogen, USA, #15140122) and 100 IU/ml IL-2 (R&D Systems, USA,
10. #202-IL-500; BRB Preclinical Biologics Repository at NCI). By day 13, all feeder cells were gone as determined
11. by flow cytometric analysis with double staining using FITC anti-CD56 (Biolegend, USA, #318304) and PE anti-
12. CD235a (Biolegend, USA, #349105) (Fig. S2a, right panel). NK-cell lines were provided by Dr. Norio Shimuzu and
13. authenticated by short tandem repeat (STR) analysis and cultured in RPMI 1640 (Thermo Fisher Scientific, USA,
14. #11-875-101) with 10% FBS, 1% (v/v) penicillin-streptomycin solution, 100 IU/ml IL-2. When indicated, AP-1
15. inhibitor T5224 (MedChemExpress, USA, #HY-12270) was added to NK-cell culture at 5 μM.

18

# NK-cell genetic modification by CRISPR/Cas9 and lentiviral transduction

1. To knock out PRDM1 in resting NK-cells, two million NK-cells were washed with PBS right after enrichment,
2. electroporated with 200 pmol ribonucleoprotein (RNP), which was formed by incubating sgRNA (Synthego,
3. USA) with Cas9 protein (QB3 MacroLab, UC Berkeley) at 1:1 molar ratio, using the P3 Primary Cell 4D-
4. Nucleofector™ X Kit S (Lonza, Basel, Switzerland, Catalog #V4XP-3032) and program EH-100. Feeder cells were
5. added to the NK-cells at the end of the day. Sequences of sgRNA targeting PRDM1 exon 4 are as follows:
6. GUUGGCAGGGAUGGGCUUAA, GAAGUGGUGAAGCUCCCCUC, and CUCUCCCCGGGAGCAAAACC. Genomic DNA
7. was used for genotyping using primers 5’-CGCCCTGATTTCTGCTGATTC and 5’-
8. CATGTTATTAGTTCAAAGGGGCAG. Inference of CRISPR Edits (ICE)^2^ analysis was used to determine the
9. knockout efficiency.
10. To knock in APEX2 in frame into the PRDM1 exon 7, freshly isolated NK-cells were cultured with irradiated
11. feeder cells at 1:1 ratio for five days, electroporated with RNP and a homology directed repair template
12. (HDRT) using Human Natural Killer Cell Nucleofector Kit (Lonza, Basel, Switzerland, Catalog #VPA-1005) and
13. arm with stop codon deletion, APEX2-T2A-EGFP, SV40 stop signal, and 3’ homologous arm was generated by
14. PCR amplification of individual fragments, Gibson assembly (New England Biolabs, USA, #E2611L) and cloning
15. into a pUC19 vector. Modified cells were enriched by fluorescence-activated cell sorting.
16. SMASh inducible system was introduced into KHYG1 to overexpress PRDM1 by lentiviral transduction. Virus
17. was produced by transfecting 293-T cells with pPAX2, pMD2, and epHIV7-PRDM1-SMASh (GFP-expressing)
18. plasmids. Supernatant were collected and concentrated by ultracentrifugation at 40,000 rcf at 4°C for 1 h 30
19. min. ~0.7 million KHYG1 cells were mixed with lentivirus and 8 μg/ml polybrene at 800 rcf at 25°C for 1 h 30
20. min. Cells were culture with 1 μM Asunaprevir (ASV) (Cayman Chemical Company, USA, #20835) to maintain a
21. low level of PRDM1 expression. To induce PRDM1 protein accumulation, cells were washed twice with PBS
22. and cultured without ASV for indicated period of time.

43

# Subcloning

1. Human PRDM1α coding sequence, SMASh (pCS6-YFP-SMASh, Addgene, USA), and T2A-EGFP sequences
2. were PCR amplified using primers NheI-PRDM1-SMASH-F: 5’- TAGAGCTAGCGAATTCGTTTAATTCGC, PRDM1a-
3. SMASH-F: 5’- GTTGAACCAATGGATCCTCCCGGGGATGAGATGGAA, PRDM1a-SMASH-R: 5’-
4. TTCCATCTCATCCCCGGGAGGATCCATTGGTTCAAC, SMASh-T2A-F: 5’-
5. GACAGGGAGGTTCTCTACTCTGGTTCTGGAGAGGGC, T2A-SMASh-R: 5’-
6. GCCCTCTCCAGAACCAGAGTAGAGAACCTCCCTGTC, and NotI-EGFP-R: 5’-
7. AGAGCGGCCGCTCACTTGTACAGCTCGTCCATGCC. These fragments were assembled by Gibson assembly kit,
8. enzyme digested, ligated to epHIV7 vector cut with the same enzymes, and transformed into Stbl2 competent
9. cells (Life Technologies, USA, #10268-019).
10. HDRT (Fig. S5a) containing 5’ homologous arm with stop codon deletion, APEX2-T2A-EGFP, SV40 stop signal,
11. and 3’ homologous arm was generated by PCR amplification of individual fragments, Gibson assembly (New
12. England Biolabs, USA, #E2611L) and cloning into a pUC19 vector. Primers used in cloning are as follows. SalI-
13. PRDM1 exon7-F: 5’- AATCGTCGACAAATCGAGAAGTTTGACATCAGTG, XbaI-PRDM1ex7-R2: 5’-
14. CATCTCTAGAGGGGCAAATATATAGACACAGGTG, Exon7-APEX2-F: 5’-
15. ATCCTGGAAGCGGCGGAGGAAAGTCTTACCCAACT, Exon7-APEX2-R: 5’-
16. AGTTGGGTAAGACTTTCCTCCGCCGCTTCCAGGAT, APEX2-HA EGFP-F: 5’-
17. TTGGGTTTGCTGATGCCGGATCGGGCGGGAGTACCGGT, APEX2-HA EGFP-R: 5’-
18. ACCGGTACTCCCGCCCGATCCGGCATCAGCAAACCCAA, GFP-ex7RHA-F: 5’-
19. TGGACGAGCTGTACAAGTAAGATTTTCAGAAAACACTTAT, GFP-ex7RHA-R: 5’-
20. ATAAGTGTTTTCTGAAAATCTTACTTGTACAGCTCGTCCA.
21. A-FOS was subcloned from, CMV500 A-FOS^3^, a gift from Charles Vinson (Addgene plasmid # 33353 ;
22. http://n2t.net/addgene:33353 ; RRID:Addgene_33353), using primers NotI-A-FOS-F 5’-
23. ATTCGCGGCCGCGAGACCCAAGCTCCACCAT and EGFP-A-FOS-R 5’-
24. GCCCTCTCCAGAACCAGAATCAGGGATCTTGCAGGC into epHIV7 backbone.
25. All constructs were sequence validated.

70

# Western blot and antibodies

1. Cells were lysed in RIPA Lysis Buffer (Santa Cruz, USA, #sc-24948A) pre-mixed with protease inhibitors.
2. Protein concentration was determined by BCA protein assay kit (Thermo Fisher Scientific, USA, #23225).
3. Protein samples were run on 8.0% SDS polyacrylamide gels and transferred to a nitrocellulose membrane (Bio-
4. Rad, USA, #1620115). The membranes were incubated with antibodies against PRDM1 (Cell Signaling, USA,
5. #9115S), TCF-1 (Cell Signaling, USA, #2203T), Bcl-11B (Cell Signaling, USA, #12120T), c-Myc (Cell Signaling, USA,
6. #5605S), c-Myb (Biolegend, USA, #935301), GAPDH antibody (Cell Signaling, USA, #2118S), NCoR1 (Cell
7. Signaling, USA, #5948S), SIN3B (Thermo Fisher Scientific, USA, #PA5-30707), TLE3 (Santa Cruz Biotechnology,
8. USA, #sc-514798), HDAC1 (Cell Signaling, USA, #5356T), G9a (Cell Signaling, USA, #3306T), EOMES (Cell
9. Signaling, USA, #4540S), and CBFβ (Novus Biologicals, USA, #H00000865-B01P)overnight at 4°C, washed and
10. incubated with peroxidase-linked anti-rabbit IgG Ab (Cell Signaling, USA, #7074S) for 1 hour at room
11. temperature, and peroxidase activity was detected by Maximum Sensitivity Substrate (Thermo Fisher
12. Scientific, USA, #34095). For visualization, films (Thermo Fisher Scientific, USA, #34090) were developed either
13. manually or using a computerized imaging system (ChemiDoc MP; Bio-Rad, USA, #12003154) was adopted.
14. The intensity of bands was quantified by densitometry when indicated.

86

# Flow cytometry

1. Cells were washed with PBS, blocked with Fc Receptor Binding Inhibitor Polyclonal Antibody (Thermo Fisher
2. Scientific, USA, # 14-9161-73), stained with surface markers, FITC-CD3 (Biolegend, USA, #300306), APC-CD56
3. (Biolegend, USA, #318310), PE-CD235a (Biolegend, USA, #349105), PE-Cy7-CD69 (Biolegend, USA, #310912),
4. PE-Cy7-CD62L (Biolegend, USA, #304822), APC-Cy7-Tim3 (Biolegend, USA, #345025), or AF647-CD16
5. (Biolegend, USA, #360713) and then washed with PBS before suspension with FACS buffer for flow cytometric
6. analysis. For cytokines and granzyme B, cells were cultured with Brefeldin A (Thermo Fisher Scientific, USA, #
7. 00-4506-51) for 4h, fixed and permeabilized before staining with APC-IFNγ (Biolegend, USA, #502511), PE-Cy7-
8. TNFα (Biolegend, USA, #376210), and APC-Cy7-granzyme B (Biolegend, USA, #372228). PI cell cycle assay
9. (Thermo Fisher Scientific, USA, #P3566) and Annexin V apoptosis assay (Biolegend, USA, #640920) were done
10. following manufacturer’s protocol.

# ChIP-qPCR

1. The efficiency of ChIP was evaluated by quantitative PCR (qPCR) on IL2RA, CIITA4 and MYC5 loci as positive
2. controls and MYOD6 as a negative control. The sequences of primers used are: IL2Ra-F: 5’-TGAAAACGGGGC
3. CAGAGAAG-3’, IL2Ra-R: 5’-TGCACTTCGGGCTTTCACTA-3’; CIITA-F: 5’- GGTTCCATTGTGATCATCA-3’, CIITA-R: 5’-
4. AAACTCTCCCTGCAAGGTG-3’; MYC-F: 5’-CAGTGCGTTCTCGGTGTG-3’, MYC-R: 5’-CAGCCGAGCACTCTAGCTCT-3’;
5. MYOD1-F: 5’-CCTCTTTCGGTCCCTCTTTC-3’, MYOD1-R: 5’-TTCCAAACCTCTCCAACACC-3’. The percentage of
6. input was calculated by the equation: percent of input = 2% x 2(CT of 2%Input Sample – CT of IP Sample).

# NK-cell cytotoxicity assay

1. NK-cells were seeded with irradiated luciferase-expressing K562 cells in triplicate in a white 96-well plate at
2. 2:1, 1:1, 0.5:1, and 0.25:1 effector-to-target ratios. After overnight coculture, 50 μl of Luciferase Assay
3. Reagent II (LAR II) (Promega, USA, #E1960) was added to each well before gentle shaking and luminescence
4. reading. Percentage of lysis was calculated based on readings of the well with target cells only.

# Chromatin-Immunoprecipitation sequencing (ChIP-seq)

1. Chromatin was prepared and immunoprecipitated using the enzymatic ChIP Kit (Cell Signaling, Magnetic
2. Beads, #9005), according to the manufacturer's instructions. A specific antibody against PRDM1 (C14A4, Cell
3. Signaling Technology, USA, #9115) was used for ChIP and normal rabbit IgG (Cell Signaling Technology, USA,
4. #2729) was used as negative control. Approximately 106 cells were used for each immunoprecipitation
5. reaction (either PRDM1 antibody or IgG). Briefly, cells were fixed with 1% formaldehyde for 10 min at room
6. temperature and then neutralized by 125 mM glycine. Crosslinked chromatin was then digested by
7. micrococcal nuclease to obtain DNA fragments between 150 bp and 900 bp. The DNA fragments were diluted
8. (1:5) and then incubated overnight with PRDM1 and IgG antibodies (2 μg Ab in 0.5ml fragments dilution).
9. Protein A coated magnetic beads were used to capture the PRDM1-bound chromatin and washed as
10. instructed. Crosslinked DNA was eluted from the beads for 0.5 h at 65°C and crosslinking was reversed with
11. proteinase K digestion for 3 h at 65°C. DNA was purified with the provided spin column.

# ChIP-seq Library, ATAC-seq Library preparation and illumina deep sequencing

1. Approximately 2-5 ng of PRDM1-ChIP enriched DNA and input DNA were end-repaired7, adaptor-ligated and
2. amplified with 10 cycles of PCR for library preparation, using Hyper Prep Kit (KAPA, USA, #KK8504). Size-
3. selection step was abandoned during the ATAC-seq protocol to maximize the library complexity. The
4. distribution of sequenced insert size was between 40 bp and 1 kb with a mean of ~120 bp. qPCR-based
5. method was used for quantification of our libraries. Cluster generation and sequencing were carried out using
6. the Illumina HiSeq 2500 or Novaseq 6000 system. FASTQ files were generated from readings passing quality
7. filters for ChIP-seq and ATAC-seq data analysis. The sequencing data are deposited at
8. [https://www.ncbi.nlm.nih.gov/sra/PRJNA1121024.](https://www.ncbi.nlm.nih.gov/sra/PRJNA1121024) Identifier: PRJNA1121024.

136

# ChIP-seq data analysis

1. Paired-end sequences of 100bp length were trimmed by Trimmomatic (v0.39) with “LEADING:10 TRAILING:10
2. SLIDINGWINDOW:4:15 MINLEN:50” parameters^8^ and aligned to hg38 genome sequence from NCBI (GenBank
3. Accession number: GCA_000001405.15) using bowtie2 (v2.3.5.1)^9^. The bam files were then sorted with
4. Samtools (v1.9)^10^ and duplicate reads were marked with GATK (v4.2.2)^11^. Unmapped reads, reads with
5. unmapped mates, multi-mapped reads, duplicate reads, low-quality reads, reads mapped within ENCODE
6. blacklist regions (v3) were filtered out using Samtools and a custom script. Reads with more than 4
7. mismatches or those with mates mapped over 2kb away were also removed with Bamtools (v2.5.1)^12^. Peak
8. calling for each sample was performed with matched input control using MACS2 (v2.2.7.1)^13^. Peaks with q-
9. score < 0.05 and score > 110 were accepted as statistically significant. Peaks were restricted to +/- 100 bp
10. around each summit. Consensus peaks were then made with bedtools (v2.30.0) by merging peaks of all
11. samples within the same group and removing those peaks that only exist in one sample^14^. Peaks were
12. annotated with nearby genes and genome regions with Homer^15^. For motif discovery, motif enrichment
13. analysis was first performed with MEME-ChIP ([v](http://meme.nbcr.net/)5.3.0)^16^, and the motif sequences identified with FIMO were
14. used to identify peaks containing PRDM1 and/or RUNX motif^17^. Gene ontology and KEGG pathway enrichment
15. of the consensus peaks were analyzed using Homer^15^. Heatmap of peak enrichment in different regions were
16. plotted using deeptools (v3.5.0)^18^. Principal component analysis was performed with the count matrix mapped
17. within the merged consensus peaks obtained using featureCounts (v2.0.1) in Subread package^19^. Dot plots,
18. UpSet plots, and bar plots were made using R packages ggplot2 (v3.4.2)^20^ and UpSetR (v1.4.0)^21^.

156

# Assay for transposase-accessible chromatin sequencing (ATAC-seq)

1. A previously published OMNI ATAC-Seq protocol was used for cell lysis, DNA tagmentation, and DNA
2. purification^22,23^. The Tn5 treated DNA was amplified with 10 cycles of PCR. 1.8X AmpurXP beads purification
3. was used for the PCR product cleanup. The libraries were validated with Agilent Bioanalyzer DNA High
4. Sensitivity Kit and quantified with qPCR.
5. Mapping and filtering of 100 bp paired-end reads were performed with Bowtie2, samtools and bamtools in
6. the same way as ChIP-seq data. Peak calling was also performed using MACS2 with q-score < 0.05 and peak
7. score > 110 and annotated with Homer with hg38. Consensus peaks were obtained with bedtools by merging
8. peaks of all samples within the same group and removing those peaks that only exist in one sample. Read
9. numbers mapped to consensus peaks were counted by featureCounts^24^. Differential peak analysis was then
10. performed with DESeq2 following the same process as RNA-seq analysis. Motif analysis with the differential
11. ATAC peaks were performed with STREME in the MEME Suite^25^. KEGG pathway enrichment was performed
12. with Homer. TOBIAS (v0.14.0)^26^ was used for motif footprints analysis based on merged bam files of all
13. samples in each group and consensus peak containing overlapping peaks between at least two samples in
14. each group. Overlapping peaks between ATAC-seq and ChIP-seq were obtained using bedtools intersect
15. function, and Venn diagrams were plotted with R package VennDiagram (v1.7.3)^27^.

173

# Quantitative PCR assay (qPCR) and Hotstart PCR assay

1. RNA was reverse transcribed using SuperScript III First-Strand Synthesis SuperMix (Thermo Fisher Scientific,
2. USA, #11752050). qPCR was performed using KAPA SYBR FAST qPCR Master Mix (Thermo Fisher Scientific,
3. USA, #7959567001). Primer sequences for qPCR are listed below.

| Primer name | Sequence (5' to 3') |
| --- | --- |
| TCF7-F | CCTGCGGATATAGACAGCACTT |
| TCF7-R | AGGTACACCAGAACCTAGCATCA |
| MYB-F | AGCCCACTGTTAACAACGACTATT |
| MYB-R | GGCTGAGGGACATTGACTATATTT |
| hBCL11B-F | TCCAGCTACATTTGCACAACA |
| hBCL11B-R | GCTCCAGGTAGATGCGGAAG |
| hGZMB-F | CCCTGGGAAAACACTCACACA |
| hGZMB-R | GCACAACTCAATGGTACTGTCG |
| hSELL-F | TGCCGAGACAATTACACAGATTT |
| hSELL-R | TGAAAGGCAGAGTCTTCTCCAG |
| hIFNG-F | TCGGTAACTGACTTGAATGTCCA |
| hIFNG-R | TCGCTTCCCTGTTTTAGCTGC |
| hMYC-F | TCCCTCCACTCGGAAGGAC |
| hMYC-R | CTGGTGCATTTTCGGTTGTTG |
| hBCL6-F | ACACATCTCGGCTCAATTTGC |
| hBCL6-R | AGTGTCCACAACATGCTCCAT |
| hRPL13A-F | ACCGTCTCAAGGTGTTTGACG |
| hRPL13A-R | GTACTTCCAGCCAACCTCGTG |
| hPRDM1-F | ACATGACCGGCTACAAGACC |
| hPRDM1-R | CCCTTGTTGCAAGTCTGACA |

178

1. Primers were designed for evaluation of the RNA level of previously described splice variant of PRDM1α,
2. PRDM1β and PRDM1 with deletion of exon 6 (PRDM1∆exon6)^28^. According to our previous findings, NKYS was
3. used as positive control, and KHYG1 was used as a negative control^29^. Total RNA was isolated from cell
4. samples using RNeasy kit (Qiagen, USA, #74106) according to the manufacturer’s instructions, with additional
5. DNase I treatment for 10 min. Eluted RNA was converted to cDNA using Superscript III Reverse Transcriptase
6. (Life Technologies, USA, #4387406). PCR was carried out to differentiate transcript with or without exon 6,
7. using cDNA from same samples and HotStarTaq Master Mix (Qiagen, German, #203443). Amplification
8. products for the PRDM1 mRNA with exon6 should be 277 bp and 148 bp for the described isoform human
9. splice variant PRDM1∆exon 6. PCR products were run on an agarose gel for visualization and comparison.
10. Sequences of primers used were as follows, PRDM1∆exon6-R 5’-GATTGCTGGTGCTGCTAAATC-3’,
11. PRDM1∆exon6-F 5’-AGAAACATGACCGGCTACAA-3’.

190

# RNA-seq

1. For RNA-seq, RNeasy Mini kit (Qiagen, USA, #74106) was used for total RNA extraction. 100 ng of total RNA
2. from each sample was used for polyadenylated RNA enrichment with oligo-dT magnetic beads, and the poly
3. (A) RNA was fragmented with divalent cations under elevated temperature. First-strand cDNA was synthesized
4. from the fragmented RNA by reverse transcription. After second-strand cDNA synthesis, the double-stranded
5. cDNA underwent end repairing, 3′ end adenylation, and ligation to bar-coded Illumina adaptors. 12 cycles of
6. PCR were performed to produce the final sequencing library. Clustering and sequencing of library templates
7. were performed on Illumina HiSeq 2500 or NovaSeq 6000 platform according to the manufacturer’s
8. guidelines.
9. RNA-seq reads of 100 bp were trimmed using Trimmomatic with options “ILLUMINACLIP: TruSeq3-
10. PE.fa:2:30:10 SLIDINGWINDOW:4:15 MINLEN:36” and mapped to hg38 reference genome with STAR^30^ and
11. reads number mapped to each gene was counted with featureCounts. Read counts were filtered to keep only
12. those with more than 5 reads in at least 3 samples and were then normalized with variance stabilizing
13. transformation. Differential expression analysis was performed using DESeq2 package (v1.28.1)^31^. In all RNA-
14. seq contrasts, genes were identified as differentially expressed if the absolute value of fold change is above
15. 1.5 and adjusted P value is lower than 0.05. Transcriptional factor activity was predicted using decoupleR
16. (v2.9.7) package^32^ based on DEGs with 200 permutations and at least 50 targets in the gene list. Differential
17. expression analysis results were integrated with ChIP-seq peaks of PRDM1 using Homer. Gene lists with fold
18. changes were used as pre-ranked gene list for Gene Set Enrichment Analysis (v4.3.2)^33^ based on hallmark gene
19. sets, selected gene sets in C7:immunologic signature, and custom gene set in [GSE112813.](https://www.ncbi.nlm.nih.gov/geo/query/acc.cgi?acc=GSE112813) Volcano plots,
20. stacked bar plots, and box plots were made with ggplot2. Heatmaps of selected genes were made with R
21. packages pheatmap (v1.0.12) and ComplexHeatmap (v2.4.3)^34^.

213

# PRDM1 isoform characterization by immunoprecipitation mass spectromApproximately 5×10^7^ NKYS cells were harvested for each sample and all steps were carried out at 4°C or on ice

1. before trypsin digestion overnight. Samples were washed once with ice-cold PBS and 0.5 ml ice-cold 1X cell
2. lysis buffer (Cell signaling, USA, #9803) was added to the cell pellet and incubated for 10 min. After brief
3. sonication (4% Input, 10 s pulse and 1 min break for 3 cycles) and centrifugation at 14,000 rcf for 10 min. The
4. supernatant was transferred and 50 μl Protein A agarose beads (Sigma-Aldrich, USA, #11134515001) were
5. added for 1 h Incubation. The pre-cleared lysate was collected for immunoprecipitation using PRDM1 (C14A4,
6. Cell Signaling, USA, Cat#9115) antibody for overnight incubation on a rotator. 50 μl protein A agarose beads
7. were then added and incubated for 4 h on a rotator. The agarose beads with antibody-bound complex were
8. spun down and washed five times with 0.5 ml 1X cell lysis buffer. The washed agarose beads were
9. resuspended with 20 μl loading buffer (4X, Invitrogen, USA, #BN2003) and heated at 100°C for 10 min. The
10. supernatant was loaded into duplicate wells on an SDS-PAGE gel (8.0%). After electrophoresis, one gel lane
11. was used for Western blot with PRDM1 antibody for visualization and localization of the PRDM1 isoforms. The
12. other gel lane was washed with ultrapure water and stained by Coomassie blue (Thermo Scientific, USA,
13. #24590) based on manufacturer’s instruction to visualize appropriate protein bands. In-Gel digestion and
14. extraction steps were carried out exactly as reported^35,36^, except that trypsin digestion was carried out using
15. recombinant LysC at 10ng/µL (Boehringer Ingelheim).
16. Mass Spectrometry data were acquired on an Orbitrap Lumos Tribrid mass spectrometer (Thermo Fisher
17. Scientific) coupled to an Ultimate 3000 ultra-high performance liquid chromatography (UHPLC) system
18. (Thermo Fisher Scientific) running binary solvent system A (LC-MS grade water, 0.1% formic acid) and B (LC-MS
19. grade acetonitrile, 0.1% formic acid). Peptides were loaded onto a trapping column (Acclaim PepMap C_18_, 75
20. µm inner diameter x 2 cm, 2 µm particle size, 100 Å pore size) using 100% solvent A for 7 min at 5µL/min.
21. After trapping, peptides were separated using a C18 analytical column (EasySpray ES802A, 75 μm inner
22. diameter × 25 cm, 2 μm particle size, 100 Å pore size) kept at 45°C, at a flow rate of 300 nL/min, using a
23. gradient of 60 min: 3% to 7% B in 5 min, 7% to 28% B in 40 min, 28% to 50% B in 5 min, 50% to 90% B in 1 min,
24. plateau at 90% B for 3 min, return to initial conditions in 1 min, and re-equilibration for 5 min. The mass
25. spectrometer was operated with a spray voltage of 2300 V and an ion transfer temperature of 275°C,
26. alternating between a data-dependent top speed mode and a parallel reaction monitoring (PRM) mode. The
27. data-dependent top speed mode acquired a survey scan in the orbitrap at a resolution of 120,000, scan range
28. of 375–1,500 *m/z*, automatic gain control (AGC) target at 400,000 normalized at 100%, and maximum ion
29. injection time at 50 ms. Every survey scan was followed by a daughter scan using collision induced dissociation
30. (CID) of top abundant peaks and detection in the ion trap with the following settings: quadrupole isolation
31. mode enabled, isolation window at 0.7 *m/z*, AGC target at 2,000 normalized at 20% with maximum ion
32. injection time 35 ms, and CID collision energy of 35%. The PRM mode acquired electron transfer dissociatiowith supplemental activation (EThcD) spectra of peptides specific to the longer isoforms
33. (YFWRIYSRGELHHFIDGFNEEK, targeting z=2-6) and the PRDM1β isoform (IYSRGELHHFIDGFNEEK, targeting z=2-
34. 5) using the following settings: spectra acquired in the orbitrap at 30,000 resolution, isolation window at 1.6
35. m/z, AGC target 50,000 normalized to 100%, automatic ETD reaction time, and 15% supplemental high-energy
36. collisional dissociation (HCD) activation.

253

# Rapid immunoprecipitation mass spectrometry of endogenous proteins (RIME) assay for analysis of

1. ***chromatin associated complexes***
2. We employed the RIME^37^ assay to detect proteins interacting with PRDM1 at the chromatin, by digestion of
3. the protein complexes pulled down by PRDM1 ChIP for further proteomic analyses. A total of 2×10^8^ NK-cells
4. were used for every RIME assay. The ChIP procedures were performed as described above with 10 μg antibody
5. in 2.5 ml diluted sample.
6. The RIME assay was then carried out as previously described^37^. Magnetic beads coated with PRDM1 antibody
7. were incubated with DNA/protein complex for 4h at 4°C, the beads were extensively washed with 1 ml of RIPA
8. buffer (50 mM HEPES (pH 7.6), 1 mM EDTA, 0.7% sodium deoxycholate, 1% NP-40, 0.5 M LiCl) at 4°C for 10
9. times. After two additional wash steps in 1 ml ice cold 100 mM ammonium bicarbonate (AMBIC), the beads
10. were incubated with 15 μL (10 ng/ul) of trypsin in 100mM AMBIC overnight at 37°C. 10 μl of trypsin buffer
11. were added to each sample for additional digestion for 4 h at 37°C. 1.3 μl formic acid was directly added to the
12. collected supernatant (~25 μl) to reach a final concentration of 5% (v/v).
13. C18 Spin Columns (Thermo Scientific, USA, #89870) were used for peptides clean-up. Cartridge was pre-
14. conditioned twice with 200 μl of 50% (v/v) acetonitrile (ACN)/water and equilibrated twice with 200 μl of 0.1%
15. (v/v) formic acid. The peptides were then loaded and re-loaded onto the cartridge with a receiver tube by
16. centrifugation and washed 4 times with 200 μL 0.1% (v/v) formic acid. Peptides bound to the resin were eluted
17. twice with 50 μl of 60% (v/v) ACN/0.1% (v/v) formic acid in a new receiver tube. All centrifugation steps were
18. carried out at 1,500 rcf for 1 min. Eluted peptides were combined and dried by speed vacuum for further
19. Liquid chromatography-mass spectrometry (LC-MS) analysis.
20. Each sample was reconstituted in 10 μl of 0.1% (v/v) formic acid and cooled to 4°C in the LC autosampler for
21. High Performance Liquid Chromatography (HPLC) and MS analysis using parameters as described^37^. Analytical
22. column temperature was set at 40°C. 5 μl of each sample was typically injected at a flow rate of 300 nl/min.
23. The eluted peptides were sprayed directly into the spectrometer for peptide ion fragmentation using collision-
24. induced dissociation.
25. The acquired raw data was further processed using Proteome Discoverer (v1.4) and Mascot and/or SEQUEST
26. as search engines. A database concatenated with a decoy database (the amino acid sequence order is either
27. randomized or reversed) was used. Decoy hits are used to estimate the false positive rate; and a false positive
28. rate of 1% was used as a cutoff for peptide identifications. Typical parameters used for searching RIME data
29. with Proteome Discoverer were adopted as described^37^. And the output comprises lists of confidently
30. identified proteins including accession numbers, protein descriptions, peptide identifications and associated
31. search statistics.

286

# APEX2 proximity-based biotinylation assay

1. Freshly isolated human NK-cells were stimulated with irradiated feeder cells in the presence of 100 u/ml IL-2
2. for five days before electroporation of Cas9/sgPRDM1 exon7 RNP and HDRT to knock in APEX2 as a fusion tag
3. at the C’-terminus of PRDM1 followed by T2A-GFP sequence GFP+ cells were sorted and cultured with feeder
4. stimulation for further study. Biotin labeling was carried out following a published protocol (Ref). Unmodified
5. (Cas9) or APEX2-modified primary human NK-cells were cultured with 5 mM biotin phenol (Sigma-Aldrich,
6. USA, #SML2135) with indicated concentration in normal culture medium for 0.5-2 h in an incubator. Cells were
7. washed twice with PBS++ (0.5 mM MgCl_2_, 1 mM CaCl_2_) and biotin labeling induced by adding 0.5 mM H_2_O_2_
8. (Sigma-Aldrich, USA, #H1009) for 1 min at room temperature and the reaction was immediately stopped by
9. adding ice-cold 2X stop/wash buffer [PBS, 0.5 mM MgCl_2_, 1 mM CaCl_2_, 10 mM Trolox (Sigma-Aldrich, USA, #
10. 238813), 20 mM sodium ascorbate (Sigma-Aldrich, USA, # A4034), 20 mM sodium azide (Sigma-Aldrich, USA, #
11. S2002)] and placed on ice. Cells were then washed twice with ice-cold 1X stop/wash buffer (PBS, 0.5 mM
12. MgCl_2_, 1 mM CaCl_2_, 5 mM Trolox, 10 mM sodium ascorbate, 10 mM sodium azide). Nuclear extract was
13. prepared by first lysing the cells with Buffer A (from SimpleChIP kit) on ice for 10 min. Nuclei were pelleted by
14. 850 rcf for 5 min at 4°C and then lysed with RIPA lysis buffer (50 mM Tris pH 8, 150 mM NaCl, 5 mM EDTA,
15. 0.5% sodium deoxycholate, 0.1% SDS, 1% Triton X-100, 1X protease inhibitor cocktail, 5 mM Trolox, 10 mM
16. sodium ascorbate, 10 mM sodium azide) on ice for 40 min with occasional vortexing. Nuclear lysate was
17. sonicated at 4% amplitude for 1 min total (6 s + 20 s break for 10 cycles). Cleared nuclear extract was
18. incubated with streptavidin Sepharose beads pre-washed with RIPA buffer for 4 h at 4°C on a rotator. Beads
19. were washed twice with RIPA, and then with 1 M KCl, 0.1 M Na_2_CO_3_, 2 M Urea in 10 mM Tris pH 8, RIPA, and
20. RIPA without detergent. For Western blotting, 60 ul elution buffer (2X LDS loading buffer, 1 M DTT, 50 mM
21. biotin solution) was added followed by incubation at 95°C for 15 min.
22. Mass spectrometry were performed using the single-pot, solid-phase-enhanced sample preparation (SP3)
23. method (PubMed ID: 30464214, 33335793). Briefly, the beads were resuspended in 400 µL of 62.5 mM Tris-
24. HCl, 1% SDS, then incubated for 5 min in 95 ℃, and centrifuged for 10 min at 13,000 rpm. The supernatant
25. was collected, and the beads were washed with 100 µL of 62.5 mM TrisHCl, 1% SDS, and centrifuged for 10
26. min at 13,000 rpm. The resulting supernatant was combined with the supernatant from the step. The total
27. protein content was measured using the Pierce BCA assay (Thermo Fisher), and 40 µg of protein per sample
28. was processed. Proteins were first reduced with 5 mM dithiothreitol (DTT), and then alkylated using 20 mM
29. iodoacetamide (IAA). Samples were then subjected to SP3 clean-up using an equal mixture of rinsed Sera-Mag
30. beads (GE Healthcare, catalog nos. 45152105050250 and 65152105050250) at a final concentration of 1
31. μg/μL. Following addition of ethanol (final concentration 50%), samples were briefly incubated with shaking
32. and then placed on a magnetic rack to remove the supernatant. The beads were rinsed twice with 80%
33. ethanol, and the supernatant was discarded. Beads were resuspended in 50 mM ammonium bicarbonate and
34. digested overnight with trypsin (Trypsin Gold, MS grade, Promega) on a ThermoMixer (Eppendorf), at 37°C
35. and 1,000 rpm. On the following day, the samples were acidified with 0.1% TFA and centrifuged at 20,000 × *g*
36. for 1 min. The peptide-containing supernatant was transferred into fresh tubes and vacuum-concentrated to
37. dryness. Dried peptides were then reconstituted in 2% acetonitrile, 0.1% formic acid containing 25 fmoles of
38. Pierce peptide retention time calibration (PRTC) mixture. MS data were acquired on an Orbitrap Lumos Tribrid
39. mass spectrometer (Thermo Fisher Scientific) coupled to an Ultimate 3000 ultra high-performance liquid
40. chromatography (UHPLC) system (Thermo Fisher Scientific) running binary solvent system A (LC-MS grade
41. water, 0.1% formic acid) and B (LC-MS grade acetonitrile, 0.1% formic acid). Peptides were injected directly (5
42. μL, 18.3 min loading time) on a C18 analytical column (EasySpray ES802A, 75 μm inner diameter × 25 cm, 2-μm
43. particle size, 100 Å pore size) kept at 45°C, and separated at a flow rate of 300 nL/min using a gradient of 120
44. min: 3% to 5% B in 11 min, 5% to 19% B in 69 min, 19% to 30% B in 20 min, 30% to 98% B in 5 min, plateau at
45. 98% B for 2 min, return to initial conditions in 1 min, and re-equilibration for 12 min. All MS data were
46. acquired in data-dependent top speed mode with the following settings: spray voltage of 2,000 V, ion transfer
47. tube temperature of 275°C, survey scan in the Orbitrap Lumos mass spectrometer at a resolution of 120,000,
48. scan range of 400–1,600 *m/z*, automatic gain control (AGC) target at 400,000 normalized at 100%, and
49. maximum ion injection time at 50 ms. Every parent scan was followed by a daughter scan using high-energy
50. collisional dissociation (HCD) of top abundant peaks and detection in the ion trap with the following settings:
51. quadrupole isolation mode enabled, isolation window at 1.6 *m/z*, AGC target at 5,000 normalized at 50% with
52. maximum ion injection time 35 ms, and HCD collision energy of 35%. Mass spectra were queried against the
53. human protein database (Swissprot, 2020) using the Byonic plugin (version 3.17.13) in Proteome Discoverer
54. (version 2.4.1.15), with the following parameters: 10 ppm precursor mass tolerance, 0.5 Da fragment mass
55. tolerance, tryptic peptide cleavage with up to 4 missed internal cleavage sites, methionine oxidation as a
56. variable modification, cysteine carbamidomethylation as fixed modification, and lysine and N-terminus
57. biotinylation as variable modification. PSMs and peptides were filtered to a 1% target false discovery rate
58. (FDR). Protein and peptide abundances were normalized using the vsn package in R v3.6.0.
59. **Reference**
60. 1. Somanchi, S.S., Senyukov, V.V., Denman, C.J. & Lee, D.A. Expansion, purification, and functional assessment of
61. human peripheral blood NK cells. *J Vis Exp* (2011).
62. 2. Conant, D. *et al.* Inference of CRISPR Edits from Sanger Trace Data. *CRISPR J* **5**, 123-130 (2022).
63. 3. Ahn, S. *et al.* A dominant-negative inhibitor of CREB reveals that it is a general mediator of stimulus-dependent
64. transcription of c-fos. *Mol Cell Biol* **18**, 967-77 (1998).
65. 4. Shaffer, A. *et al.* Blimp-1 orchestrates plasma cell differentiation by extinguishing the mature B cell gene
66. expression program. *Immunity* **17**, 51-62 (2002).
67. 5. Morgan, M.A., Mould, A.W., Li, L., Robertson, E.J. & Bikoff, E.K. Alternative splicing regulates Prdm1/Blimp-1
68. DNA binding activities and corepressor interactions. *Molecular and cellular biology* **32**, 3403-3413 (2012).
69. 6. Basu, A., Dasari, V., Mishra, R.K. & Khosla, S. The CpG island encompassing the promoter and first exon of
70. human DNMT3L gene is a PcG/TrX response element (PRE). *PloS one* **9**, e93561 (2014).
71. 7. Kanai, T. *et al.* Identification of STAT5A and STAT5B target genes in human T cells. *PLoS One* **9**, e86790 (2014).
72. 8. Bolger, A.M., Lohse, M. & Usadel, B. Trimmomatic: a flexible trimmer for Illumina sequence data. *Bioinformatics*

360 **30**, 2114-20 (2014).

1. 9. Langmead, B. & Salzberg, S.L. Fast gapped-read alignment with Bowtie 2. *Nat Methods* **9**, 357-9 (2012).
2. 10. Li, H. *et al.* The Sequence Alignment/Map format and SAMtools. *Bioinformatics* **25**, 2078-9 (2009).
3. 11. McKenna, A. *et al.* The Genome Analysis Toolkit: a MapReduce framework for analyzing next-generation DNA
4. sequencing data. *Genome Res* **20**, 1297-303 (2010).
5. 12. Barnett, D.W., Garrison, E.K., Quinlan, A.R., Strömberg, M.P. & Marth, G.T. BamTools: a C++ API and toolkit for
6. analyzing and managing BAM files. *Bioinformatics* **27**, 1691-2 (2011).
7. 13. Zhang, Y. *et al.* Model-based analysis of ChIP-Seq (MACS). *Genome Biol* **9**, R137 (2008).
8. 14. Quinlan, A.R. & Hall, I.M. BEDTools: a flexible suite of utilities for comparing genomic features. *Bioinformatics*

369 **26**, 841-2 (2010).

1. 15. Heinz, S. *et al.* Simple combinations of lineage-determining transcription factors prime cis-regulatory elements
2. required for macrophage and B cell identities. *Mol Cell* **38**, 576-89 (2010).
3. 16. Machanick, P. & Bailey, T.L. MEME-ChIP: motif analysis of large DNA datasets. *Bioinformatics* **27**, 1696-7 (2011).
4. 17. Grant, C.E., Bailey, T.L. & Noble, W.S. FIMO: scanning for occurrences of a given motif. *Bioinformatics* **27**, 1017-8

374 (2011).

375 18. Ramírez, F. *et al.* deepTools2: a next generation web server for deep-sequencing data analysis. *Nucleic Acids Res*

376 **44**, W160-5 (2016).

1. 19. Liao, Y., Smyth, G.K. & Shi, W. featureCounts: an efficient general purpose program for assigning sequence reads
2. to genomic features. *Bioinformatics* **30**, 923-30 (2014).
3. 20. Wickham, H. *ggplot2*
4. *Elegant Graphics for Data Analysis*, (Springer-Verlag New York, 2016).
5. 21. Conway, J.R., Lex, A. & Gehlenborg, N. UpSetR: an R package for the visualization of intersecting sets and their
6. properties. *Bioinformatics* **33**, 2938-2940 (2017).
7. 22. Corces, M.R. *et al.* An improved ATAC-seq protocol reduces background and enables interrogation of frozen

384 tissues. *Nat Methods* **14**, 959-962 (2017).

385 23. Grandi, F.C., Modi, H., Kampman, L. & Corces, M.R. Chromatin accessibility profiling by ATAC-seq. *Nat Protoc* **17**,

386 1518-1552 (2022).

1. 24. Liao, Y., Smyth, G.K. & Shi, W. The Subread aligner: fast, accurate and scalable read mapping by seed-and-vote.
2. *Nucleic Acids Res* **41**, e108 (2013).
3. 25. Bailey, T.L. STREME: accurate and versatile sequence motif discovery. *Bioinformatics* **37**, 2834-2840 (2021).
4. 26. Bentsen, M. *et al.* ATAC-seq footprinting unravels kinetics of transcription factor binding during zygotic genome
5. activation. *Nat Commun* **11**, 4267 (2020).
6. 27. Chen, H. & Boutros, P.C. VennDiagram: a package for the generation of highly-customizable Venn and Euler
7. diagrams in R. *BMC Bioinformatics* **12**, 35 (2011).
8. 28. Smith, M.A. *et al.* PRDM1/Blimp-1 controls effector cytokine production in human NK cells. *The Journal of*

395 *Immunology* **185**, 6058-6067 (2010).

396 29. Küçük, C. *et al.* PRDM1 is a tumor suppressor gene in natural killer cell malignancies. *Proceedings of the National*

397 *Academy of Sciences* **108**, 20119-20124 (2011).

1. 30. Dobin, A. *et al.* STAR: ultrafast universal RNA-seq aligner. *Bioinformatics* **29**, 15-21 (2013).
2. 31. Love, M.I., Huber, W. & Anders, S. Moderated estimation of fold change and dispersion for RNA-seq data with
3. DESeq2. *Genome Biol* **15**, 550 (2014).
4. 32. Badia, I.M.P. *et al.* decoupleR: ensemble of computational methods to infer biological activities from omics data.
5. *Bioinform Adv* **2**, vbac016 (2022).
6. 33. Subramanian, A. *et al.* Gene set enrichment analysis: a knowledge-based approach for interpreting genome-
7. wide expression profiles. *Proc Natl Acad Sci U S A* **102**, 15545-50 (2005).
8. 34. Gu, Z., Eils, R. & Schlesner, M. Complex heatmaps reveal patterns and correlations in multidimensional genomic
9. data. *Bioinformatics* **32**, 2847-9 (2016).
10. 35. Shevchenko, A., Tomas, H., Havlis, J., Olsen, J.V. & Mann, M. In-gel digestion for mass spectrometric
11. characterization of proteins and proteomes. *Nat Protoc* **1**, 2856-60 (2006).
12. 36. Vu, L. *et al.* Defining the Caprin-1 Interactome in Unstressed and Stressed Conditions. *J Proteome Res* **20**, 3165-

410 3178 (2021).

411 37. Mohammed, H. *et al.* Rapid immunoprecipitation mass spectrometry of endogenous proteins (RIME) for analysis

412 of chromatin complexes. *Nat Protoc* **11**, 316-26 (2016).

413

414

415

416

417

418

419

420

421

422

423

424

425
